# Inducing Ectopic T Cell Clusters Using Stromal Vascular Fraction Spheroid‐Based Immunotherapy to Enhance Anti‐Tumor Immunity

**DOI:** 10.1002/advs.202203842

**Published:** 2022-09-04

**Authors:** Jae‐Won Lee, Bum Chul Park, Na Yoon Jang, Sihyeon Lee, Young Kyu Cho, Prashant Sharma, Sang Won Byun, Kyeongseok Jeon, Yun‐Hui Jeon, Uni Park, Hyo Jin Ro, Hyo Ree Park, Yuri Kim, Dong‐Sup Lee, Seok Chung, Young Keun Kim, Nam‐Hyuk Cho

**Affiliations:** ^1^ Department of Biomedical Sciences Seoul National University College of Medicine Seoul 03080 Korea; ^2^ Department of Microbiology and Immunology Seoul National University College of Medicine Seoul 03080 Korea; ^3^ Institute of Endemic Diseases College of Medicine Seoul National University Seoul 03080 Korea; ^4^ Department of Materials Science and Engineering Korea University Seoul 02481 Korea; ^5^ Brain Korea Center for Smart Materials and Devices Korea University Seoul 02841 Korea; ^6^ School of Mechanical Engineering Korea University Seoul 02841 Republic of Korea; ^7^ Seoul National University Bundang Hospital Seongnam‐si Gyeonggi‐do 13620 Republic of Korea

**Keywords:** cancer immunotherapy, dendritic cells, immunotherapy, iron‐oxide zinc oxide nanoparticles, spheroid, stromal vascular fraction, tertiary lymphoid structure

## Abstract

Tertiary lymphoid structures (TLSs) provide specialized niches for immune cells, resulting in improved prognoses for patients undergoing cancer immunotherapy. Shaping TLS‐like niches may improve anti‐cancer immunity and overcome the current limitations of immune cell‐based immunotherapy. Here, it is shown that stromal vascular fraction (SVF) from adipose tissues can enhance dendritic cell (DC)‐mediated T cell immunity by inducing ectopic T lymphocyte clusters. SVF cells expanded ex vivo have phenotypes and functions similar to those of fibroblastic reticular cells in a secondary lymphoid organ, and their properties can be modulated using three‐dimensional spheroid culture and coculture with DCs spiked with antigen‐loaded iron oxide–zinc oxide core‐shell nanoparticles. Thereby, the combination of SVF spheroids and mature DCs significantly augments T cell recruitment and retention at the injection site. This strategy elicits enhanced antigen‐specific immune response and anti‐tumoral immunity in mice, illustrating the potential for a novel immunotherapeutic design using SVF as a structural scaffold for TLS.

## Graphical abstract

Expanded stromal vascular fraction (SVF) cells show similar functional characteristics to lymph node stromal cells. Coculturing of SVF cells and dendritic cells (DCs) shows reciprocal interaction to support one another and enhance T cell responses. in vivo engraftment of SVF spheroids and antigen‐loaded DCs actively induces ectopic T cell lymphoid cluster and enhance both local and systemic antigen‐specific immune responses.

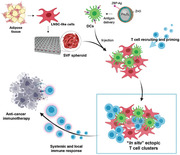

## 1 Introduction

Dendritic cells (DCs), the most potent mediators between innate and adaptive immunity, are promising agents for cancer vaccines. However, owing to insufficient clinical response rates, DC‐based products remain unavailable.^[^ ^1^, ^2^, ^3^ ^]^ To establish an antigen‐specific immune response, DCs must migrate to draining lymph nodes (LNs) to encounter T cells.^[^ ^4^ ^]^ However, only 4%–5% of DCs injected migrate to LNs, whereas most remain at the injection site and die within 48 h.^[^ ^5^, ^6^, ^7^ ^]^ Therefore, utilizing resident DCs at the injection site could enhance DC‐mediated immune response to realize the full potential of DC‐based therapy.

Specialized compartments of lymphoid organs, consisting of CD45^–^ non‐hematopoietic stromal cells, facilitate maximum interactions between T cells and DCs.^[^ ^4^ ^]^ PDPN^+^CD31^–^ fibroblastic reticular cells (FRCs) are a principal component of the lymphoid stroma.^[^ ^8^ ^]^ FRCs regulate T cell and DC homeostasis and provide structural support for immune responses through three‐dimensional (3D) networks for immune cell migration.^[^ ^9^, ^10^, ^11^, ^12^ ^]^ Hence, FRCs can be used to enhance DC‐mediated T cell responses. Nevertheless, obtaining functional stromal cells from lymphoid organs for immunotherapy is extremely challenging as they constitute less than 5% of all cells in the organs.^[^ ^13^ ^]^

Stromal cell precursors in adipose tissues can differentiate into LN stromal cells during LN development or lymphoid structure generation.^[^ ^14^, ^15^, ^16^ ^]^ Multiple studies have observed lymphoid structures in fat tissues,^[^ ^17^, ^18^ ^]^ indicating that adipose tissues can serve as an alternative source of LN stromal cells. Local fibroblasts and myeloid cells such as DCs can also alter the stromal properties of fibroblasts into those of FRCs, resulting in the recruitment and retention of immune cells and the formation of tertiary lymphoid structures (TLSs).^[^ ^19^ ^]^ TLSs have been associated with improved prognoses of cancer immunotherapy;^[^ ^20^, ^21^ ^]^ hence, induction of TLSs is a promising therapeutic strategy.^[^ ^22^, ^23^ ^]^

**Figure 1:**
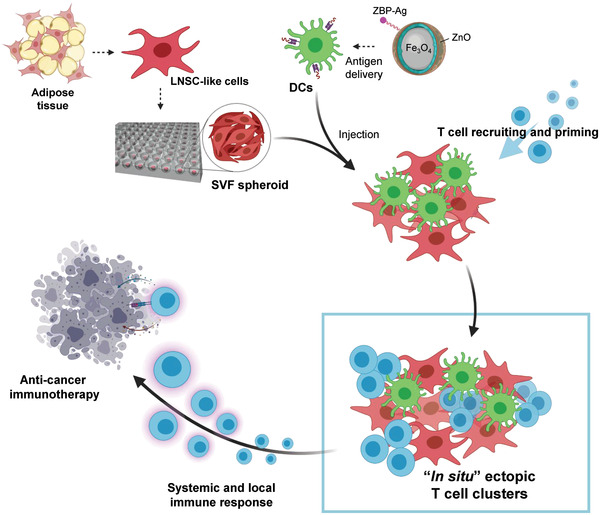
Schematic strategy for generation of in situ ectopic T cell clusters to enhance the anti‐tumor effect. Cellular engineering of stromal vascular fraction (SVF) cells and dendritic cells (DCs), using three‐dimensional spheroid culture and nanoparticles for antigen delivery, improves immune response via generation of in situ ectopic T cell clusters. LNSC‐like cells, lymph node stromal cell‐like cells.

We hypothesize that adipose tissue stromal vascular fraction (SVF) cells, instead of FRCs, could enhance resident DC‐mediated antigen‐specific immunity by providing TLS‐like niches at the injection site. Here, we describe an approach for generating in situ ectopic T lymphocyte clusters in a mouse melanoma model by manipulation of DCs and SVF cells using nanoparticle‐mediated antigen delivery^[^ ^24^, ^25^ ^]^ and 3D culture technology,^[^ ^26^ ^]^ enabling recruitment and retention of T cells at the graft site (**Figure** **1**). A combination of SVF spheroids (SPHs) and mature DCs (mDCs) exposed to ovalbumin (OVA)‐conjugated nanoparticles (NPs) significantly augmented the antigen‐specific T cell response and enhanced systemic and local immunity in a mouse melanoma model. Our strategy may facilitate the development of SVF cells as a structural scaffold toward a novel cancer immunotherapeutic design.

## 2 Results

### 2.1 Characteristics of SVF Cells Expanded from Adipose Tissues

SVF cells from mouse adipose tissue were cultured in Dulbecco's modified Eagle's Medium containing 10% fetal bovine serum and characterized using flow cytometry (Figure S1a,b, Supporting Information). CD45^+^ leukocytes were barely detected (< 5%) after 5 days of in vitro culture (Figures S1c, S2, Supporting Information), whereas CD45^–^ non‐hematopoietic cells constituted more than 95% of the cells after 5 days regardless of their passage number (Figure S1c, Supporting Information). Most of the CD45^–^ adherent cells showed fibroblastic morphology (Figure S1b, Supporting Information). Flow cytometry revealed that approximately 2% of the CD45^–^ cells were PDPN^+^ CD31^–^ (similar to FRCs^[^ ^8^ ^]^) at day 0, which increased to 80% on day 5 and further up to 95% at 10 days after the second passaging (Figure S1d,e, Supporting Information). We then analyzed the adherent CD45^–^ cells for FRC markers (Figure S1f, Supporting Information); lymphotoxin *β* receptor (LT*β*R), CD140*α*, ICAM‐1, and VCAM‐1 expression was significantly higher on the PDPN^+^CD31^–^ cells at day 5, and thereafter compared with that at day 0. Additionally, the CD45^–^ cells highly expressed mesenchymal stem cell/stromal cell markers (CD44, CD34, and Sca‐1) after in vitro culture (Figure S1g, Supporting Information).

To determine whether the PDPN^+^CD31^–^ cells have the functional properties of FRCs, mRNA expression of representative chemokines released by FRCs (*ccl19*, *ccl21*, *cxcl13*, and *cxcl12*) was measured following stimulation with the Toll‐like receptor (TLR) 3 agonist Poly I:C, TLR‐7 agonist imiquimod, or *α*‐LT*β*R antibody (Figure S1h, Supporting Information). Expression of all chemokine genes was significantly elevated upon stimulation compared with that in unstimulated cells. These results indicated that PDPN^+^CD31^–^ cells in expanded SVF cells resemble FRCs in phenotype and function.^[^ ^14^ ^]^

### 2.2 Immunogenic Hallmarks in Co‐Cultured SVF Cells and DCs

**Figure 2:**
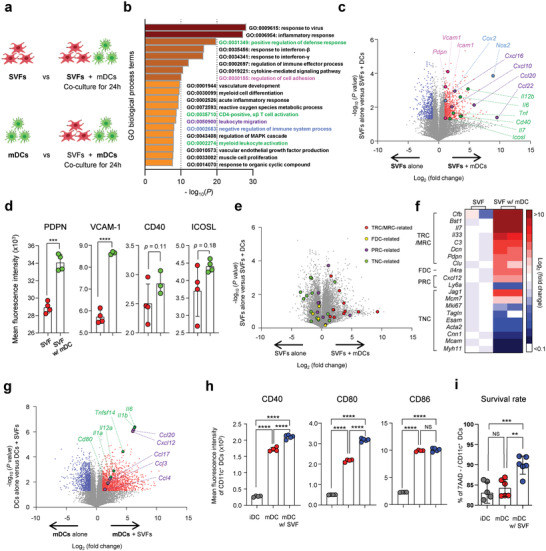
Expanded SVF cells and mDCs show upregulation of genes and molecules supporting T cell response through reciprocal interaction. a) Experimental design for transcriptome analysis. SVF cells and mature DCs were cocultured for 24 h, sorted by magnetic cell separation using anti‐CD45 microbeads, and applied in the transcriptomic analysis of each cellular subset. b) Top 20 upregulated gene sets in SVF cells cocultured with mDCs (SVFs+mDCs) for 24 h when compared with those in the cultured SVF cells (SVFs). c) Volcano plot of the transcriptome displaying upregulated genes in SVFs (blue, *p* < 0.05) or in SVFs+mDCs (red, *p* < 0.05) (*n* = 2). Representative genes in (b) are colored accordingly. d) Expression of indicated markers in SVFs and SVFs+mDCs (*n* = 3 or 4, mean ± s.e.m.). e) Differential expression of FRC subtype markers in SVFs and SVFs+mDCs. f) Heat map of significantly modulated (*p* < 0.05) genes belonging to the indicated subtypes. g) Volcano plot of transcriptome displaying upregulated genes in mDCs alone (blue, *p* < 0.05) or mDCs cocultured with SVF cells (red, *p* < 0.05) for 24 h (*n* = 2). Representative genes related to immunostimulation and chemotaxis are indicated. h) Expression of indicated markers in immature DCs (iDC), mDCs, and mDCs cocultured with SVF cells (*n* = 5, mean ± s.e.m.). i) Survival rates of CD11c^+^ DCs (*n* = 6, mean ± s.e.m.). *: *p* < 0.05, **: *p* < 0.01, ***: *p* < 0.001, **** *p*: < 0.0001, NS: nonsignificant using two‐tailed students’ *t*‐test or one‐way ANOVA with Tukey's post‐hoc test.

To show whether expanded SVF cells can support DC‐mediated immune response, SVF cells and immature DCs stimulated with lipopolysaccharides (mDCs) were incubated separately or together for 24 h and their transcriptomes were analyzed (**Figure** **2**a).

Gene ontology term enrichment revealed that genes involved in immunostimulation, cell migration, as well as immunosuppression, were significantly upregulated in SVF cells cocultured with mDCs compared with those in SVF cells cultured alone (Figure 2b and Figure S3, Supporting Information). We then focused on gene sets involved in the regulation of T cell responses (Figure 2c). In particular, genes involved in T cell activation (*il12b*, *il6*, *tnf*, *CD40, icosl*), T cell survival (*il7*), T cell chemotaxis (*ccl20*, *ccl22*, *cxcl10*, and *cxcl16*), and adhesion molecules (*icam1*, *pdpn*, and *vcam1*) were upregulated in SVF cells cocultured with mDCs compared to those in SVF cells cultured alone. Additionally, genes involved in T cell functional regulation, such as *nos2* and *cox2*, were upregulated.^[^ ^27^, ^28^ ^]^ Flow cytometry revealed significantly higher expression of PDPN and VCAM‐1 in SVF cells cocultured with mDCs than in SVF cells cultured alone. Additionally, higher mean fluorescence intensities for CD40 and ICOSL were observed in SVF cells cocultured with DCs; however, the differences were not significant (Figure 2d). When we performed principal component analysis (PCA) to compare the transcriptional signatures in SVF cells with other lymphoid organ stromal cells, coculturing with mDCs induced dramatic shifts in transcriptional profiles of SVF cells and drove them to be similar to those of lymph node FRCs and thymic fibroblasts (Figure S4a, Supporting Information).^[^ ^29^ ^]^ Hierarchical clustering analysis using differentially expressed gene sets in SVF cells cocultured with mDCs and the lymphoid organ stromal cells revealed similar gene‐expression profiles, especially in genes related to adhesion, T cell activation, and chemotaxis (Figure S4b, Supporting Information).

To further characterize expanded SVF cells in the presence or absence of DCs, we compared the transcriptional signatures of SVF cells with those of FRC subtypes of lymphoid organs: T cell zone reticular cells (TRCs), marginal reticular cells (MRCs), follicular dendritic cells (FDCs), perivascular reticular cells (PRCs), and triple‐negative cells (TNCs).^[^ ^30^ ^]^ Significant upregulation of TRC and MRC markers was observed in SVF cells cocultured with DCs, whereas TNC marker genes were upregulated in SVF cells cultured alone (Figure 2e). The SVF cells generally phenocopied TRCs and MRCs in their transcriptome when cocultured with DCs and showed reduced TNC‐related gene expression compared with SVF cells cultured without DCs (Figure 2f).

SVF cells also showed functional improvements in DCs such that genes encoding immunostimulatory factors (*cd80*, *il1a*, *il1b*, *il6*, *il12a*, and *tnfsf14*) and chemokines that attract T cells (*ccl3*, *ccl4*, *ccl17*, *ccl20*, and *cxcl12*) were upregulated in mDCs cocultured with SVF cells compared with those in DCs alone (Figure 2g). Moreover, in mDCs cocultured with SVF cells, expression of the costimulatory molecules for T cell activation, CD40, and CD80, was significantly elevated (Figure 2h). Furthermore, the survival rate of DCs after 24 h of culture was significantly improved when cocultured with SVF cells (mean: 90.0%) than when cultured alone (84.2% in mDC and 83.1% in immature DCs) (Figure 2i). These results suggest that expanded SVF cells and DCs may communicate and enhance each other's activation and survival, which may ultimately support DC‐mediated T cell immunity.

### 2.3 Formation of Immunogenic SVF Spheroids

**Figure 3:**
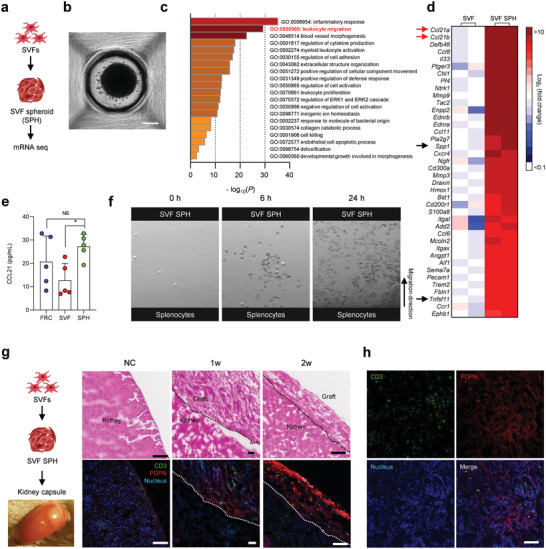
SVF spheroids (SPH) show upregulation of genes and molecules involved in T cell recruitment. a) Experimental design for transcriptomic analysis of SVF SPHs. b) Microscopic images of SVF SPHs. Scale bar: 200 µm. c) Top 20 upregulated gene sets in SVF SPHs when compared with those in 2D‐cultured SVF cells (SVF). d) Heat map of genes related to leukocyte migration in c (*p* < 0.05). e) Comparison of CCL21 expression in FRC, SVF, and SVF SPHs using ELISA (*n* = 6, mean ± s.e.m. *: *p* < 0.05). f) Cell migration assay to assess the chemotactic effect of SVF SPHs for 24 h. The black arrow represents the migration direction. g) Experimental process (left), representative H&E‐stained images (right upper panels), and immunofluorescence microscopy images (right lower panels) for normal kidney as negative control (NC) and for grafts transplanted underneath the kidney capsule after 1 or 2 weeks. Scale bar: 100 µm. h) Confocal images from the graft were obtained a week after transplantation. Scale bar: 100 µm.

3D cell culture systems mimic the in vivo cellular microenvironment to modulate gene expression and biological behavior of cells.^[^ ^31^ ^]^ To simulate the dense 3D structural environment of lymphoid tissues, we induced the formation of SVF SPHs of ≈200 µm diameter and compared their gene expression patterns with those in 2D‐cultured SVFs using transcriptome analysis (**Figure** **3**a,b). Metascape analysis revealed that the expression of gene sets related to leukocyte migration was highly increased in SVF SPHs (Figure 3c,d). Interestingly, the gene encoding the primary T cell attractant, *ccl21*, was the most upregulated in SVF SPHs, with ≈483 times higher expression than that in 2D‐cultured SVFs (Figure 3d). In addition, expression of *spp1* (osteopontin, OPN) and *tnfsf11* (RANKL), which improves the function and survival of DCs,^[^ ^32^, ^33^ ^]^ was remarkably elevated in SVF SPHs compared with that in 2D‐cultured SVF cells (Figure 3d and Figure S5a, Supporting Information).

Consistent with the higher mRNA levels, CCL21 secretion from SVF SPHs (mean: 27.2 pg ml^−1^) was approximately two‐fold higher than that from 2D‐cultured SVF cells (12.7 pg ml^−1^) and was comparable to that from FRCs (20.6 pg ml^−1^) (Figure 3e). We also confirmed the chemotactic effect of SVF SPHs on splenocytes using a cell migration assay. Active migration of the splenocytes toward SVF SPHs through penetration of the collagen matrix was observed, whereas, in the absence of SVF SPHs, there was no detectable migratory activity (Figure 3f and Movies S1, S2, Supporting Information). Further, to verify whether SVF SPHs can recruit T cells in vivo, we transplanted SVF SPHs underneath the kidney capsule in C57BL/6 mice. The grafted sites contained substantial levels of T cells, whereas T cells were barely detected in normal kidney capsules (Figure 3g,h). The results suggest that SVF cells modulated by 3D spheroid culture can recruit T cells in vivo, potentially via enhanced expression of chemotactic cytokines.

### 2.4 Induction of Ectopic T Cell Cluster In Vivo

**Figure 4:**
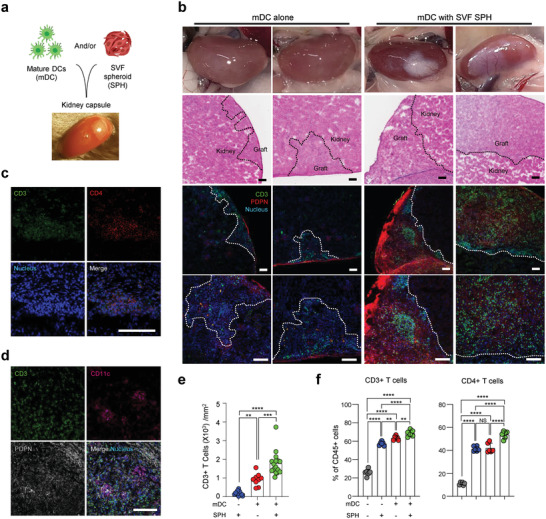
SVF SPHs and mDCs induce ectopic T cell clusters in vivo. a) Schematic illustration of in vivo experiments for induction of ectopic T cell clusters using SVF SPHs and mDCs. b) Representative images in mDC‐transplanted (left panels) and in SVF SPH+mDC‐transplanted (right panels) groups: photographic images (top row), H&E‐stained images (second row), immunofluorescent confocal images (third row), and enlarged images of those in the third row (fourth row) of the graft site of kidney tissue 2 weeks post‐transplantation. Dotted lines: the boundary between the graft and the kidney tissue. Scale bar: 100 µm c) Representative images of CD3^+^ CD4^+^ T cells in SVF SPH and mDC‐transplanted group. Scale bar: 100 µm d) Representative images of CD3^+^ T cells and CD11c^+^ DCs in SVF SPH + mDC‐transplanted group. Scale bar: 100 µm e) CD3^+^ T cell numbers determined from confocal images (cells per mm^2^) of the SVF SPH‐transplanted group (*n* = 12), mDC‐transplanted group (*n* = 9), and SVF SPH + mDC‐transplanted group (*n* = 16). mean ± s.e.m. f) Frequency of CD3^+^ T cells and CD4^+^ T cells in the indicated groups assessed using flow cytometric analysis of cells isolated from the kidneys transplanted with SVF SPHs and/or mDCs (*n* = 6, mean ± s.e.m.). *P < 0.05, **P < 0.01, ***P < 0.001, ****P < 0.0001; NS, nonsignificant using one‐way ANOVA with Tukey's post‐test.

Considering SVF cells cocultured with DCs and SVF SPHs exhibited enhanced potential for T cell recruitment, SVF SPHs and/or mDCs were transplanted in sub‐renal capsules in C57BL/6 mice to investigate whether they can recruit and modulate T cell function in vivo. (**Figure** **4**a). External traces at the cellular graft sites under the kidney capsules in SVF SPH with the mDC‐transplanted group (SVF SPH+mDC) were relatively larger than those in the mDC‐transplanted group. In addition, white patch grafts with blood vessels on the surface were observed in the SVF SPH+mDC‐transplanted (Figure 4b upper panel) at the graft site, which were barely detected in the mDC‐transplanted group. Moreover, CD3^+^ T cells were densely recruited at the graft site in the SVF SPH+mDC‐transplanted group, but barely observed in the mDC‐transplanted group (Figure 4b lower panels and Figure S6a, Supporting Information). T cell number per square millimeter was two‐fold higher in the SVF SPH+mDC‐transplanted group (mean ± S.D.: 1775.1 ± 614.9 cells mm^−2^) than in the mDC‐transplanted group (920.5 ± 316.6 cells mm^−2^) (Figure 4e). The recruited CD3^+^ T cells were primarily of the CD4^+^ T cell population, as revealed by immunohistochemistry and flow cytometry (Figure 4c,f, and Figure S6b, Supporting Information), whereas CD8^+^ T cells were rarely detected (Figure S6b, Supporting Information). Flow cytometric quantification performed on cells isolated from the whole kidney revealed that the percentage of CD3^+^ T cells among the CD45^+^ T cells recruited in the SVF SPH+mDC‐transplanted group was significantly higher than that in other groups, and the majority of those CD3^+^ T cells were CD4^+^ (Figure 4f). Further, the frequency of CD4^+^CD44^+^CD62L^–^ effector T cells in the SVF SPH+mDC‐transplanted group was significantly higher than that in other control groups (Figure S7e,f, Supporting Information). The frequency of CD4^+^FOXP3^+^ regulatory T cells was not significantly different among groups (Figure S7g, Supporting Information). We also observed CD11c^+^ DCs in contact with T cells in confocal images (Figure 4d and Figure S6c, Supporting Information). The frequencies of B cells, macrophages, and both lineages of the conventional DCs, cDC1, and cDC2, were not significantly different among groups (Figure S7b,c, Supporting Information). Overall, the results suggest that transplantation of SVF SPHs and DCs can generate in situ T cell lymphoid clusters in vivo and may facilitate interaction between T cells and the antigen‐presenting cells.

### 2.5 Enhanced Systemic and Local Antigen‐Specific T Cell Response

Next, we investigated whether the ectopic lymphoid structure formed by SVF SPHs and mDCs in the sub‐renal capsule could enhance the antigen‐specific T cell response. Ovalbumin (OVA) was complexed on the surface of 10 nm‐sized Fe_3_O_4_–ZnO NPs mediated by zinc oxide‐binding peptide (ZBP) to facilitate enhanced antigen uptake into DCs as shown in our previous study (Figure S8, Supporting Information).^[^ ^24^ ^]^ We immunized C57BL/6 mice with SVF SPHs and/or NP‐OVA‐pulsed DCs (antigen‐pulsed mDCs, amDCs) via sub‐renal capsule transplantation twice with a one‐week interval.

**Figure 5:**
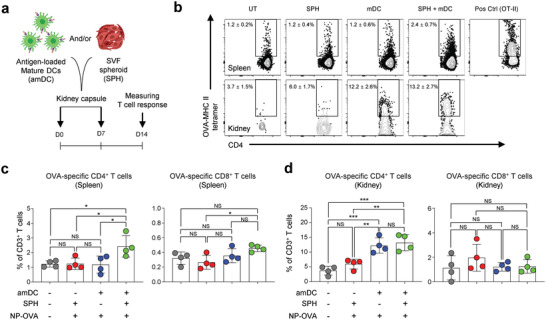
SVF SPH and antigen‐loaded amDCs enhance antigen‐specific T cell response in both systemic and local immunity. a) Experimental design of immunization with SVF SPHs and antigen‐loaded amDCs via sub‐renal capsule transplantation. b) Representative dot plots of OVA‐specific CD4^+^ T cells stained with peptide‐loaded MHC tetramers in the spleen (top) and the kidney (bottom). Untreated group (UT); SVF SPH (SPH)‐, amDC‐, and SVF SPH + amDC‐transplanted groups; and OT‐II mouse splenocytes as the positive control (Pos Ctrl (OT‐II)). c) Frequency of OVA‐specific CD4^+^ and CD8^+^ T cells in the indicated groups, in the spleen to represent systemic immunity. d) Frequency of OVA‐specific CD4^+^ and CD8^+^ T cells in the kidney, to represent local immunity, in the indicated groups (*n* = 4, mean ± s.e.m.). *: *p* < 0.05, **: *p* < 0.01, ***: *p* < 0.001; NS, nonsignificant using one‐way ANOVA with Tukey's post‐test (c and d).

To evaluate the systemic and local immune response, the antigen‐specific T cell response in the spleen and kidney was measured (**Figure** **5**a). The percentage of OVA‐specific CD4^+^ T cells in the spleen of mice immunized with SVF SPH+amDC transplantation (2.4 ± 0.7% of CD3^+^ T cells) was significantly higher than that in control groups (1.2 ± 0.2% in untreated, 1.2 ± 0.4% in SVF SPH‐transplanted, and 1.2 ± 0.6% in amDC‐transplanted groups) (Figure 5b,c, and Figure S9, Supporting Information). In the kidney, the frequency of OVA‐specific CD4^+^ T cells was significantly higher in amDC‐transplanted (12.2 ± 2.6% of CD3^+^ T cells) and SVF SPH+amDC‐transplanted (13.2 ± 2.7%) groups than in the remaining control groups (3.7 ± 1.5% in untreated and 6.0 ± 1.7% in SVF SPH‐transplanted groups). For OVA‐specific CD8^+^ T cells, although no significant differences in the percentage of OVA‐specific CD8^+^ T cells in the kidneys were seen among groups, a significantly higher percentage was observed in the spleen of the SVF SPH+amDC‐transplanted group than in that of the SVF SPH‐transplanted group (Figure 5c,d). The results indicate that SVF SPHs and DCs pulsed with NP‐OVA can further enhance the systemic T cell response when compared with DCs alone, and can generate local T cell immunity in an antigen‐dependent manner.

### 2.6 Enhancement of DC‐Mediated Anti‐Tumor Immunity by SVF SPHs

**Figure 6:**
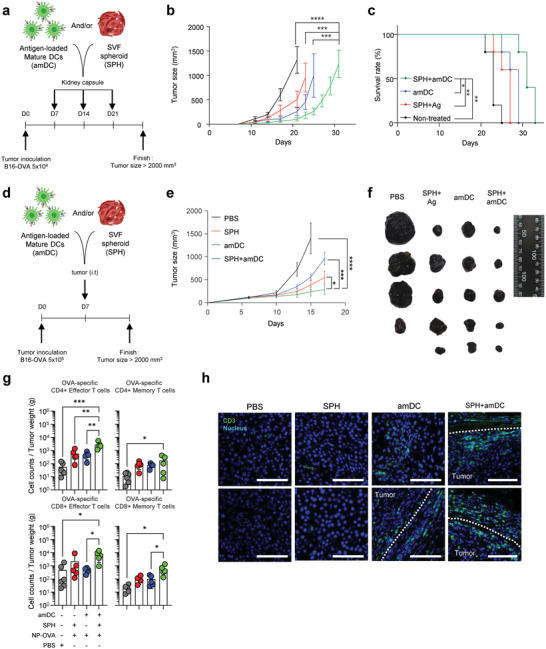
SVF SPHs and amDCs exhibit enhanced anti‐tumor effects. a) Experimental scheme for immunization with SVF SPHs and amDCs in mice bearing a tumor (OVA‐expressing B16 melanoma) via sub‐renal capsule transplantation. b) Tumor progression (*n* = 8 per group, mean ± s.e.m.). c) Survival curves (*n* = 5 per group). d) Experimental scheme for intratumoral injection (i.t) of SVF SPHs and amDCs in mice bearing a tumor (OVA‐expressing B16 melanoma). e) Tumor growth curves (*n* = 9 to 10/group, mean ± s.e.m.) f–h) Tumors on day 15–17 were used for flow cytometric analysis and IF, separately. f) Representative images of dissected tumors from the mice group on day 15. g) Number of tumor‐infiltrating OVA‐specific CD4^+^ effector (CD44^+^ CD62L^–^) and memory (CD44^+^ CD62L^+^) T cells (top row) and CD8^+^ effector T cells (CD44^+^ CD62L^–^) and memory (CD44^+^ CD62L^+^) T cells (bottom row) per gram of tumor weight (*n* = 5, mean ± s.e.m.). h) Confocal images of infiltrating CD3^+^ T cells in the tumor. The white dotted line represents the boundary of the tumor. Scale bar: 100 µm. *: *p* < 0.05, **: *p* < 0.01, ***: *p* < 0.001; NS, nonsignificant using two‐way ANOVA with Tukey's post‐test (b and e), Mantel‐Cox test (c), and one‐way ANOVA with Tukey's post‐test (g). All experiments were repeated twice.

To determine whether SVF SPH+amDC immunization elicits effective anti‐tumor responses, C57BL/6 mice inoculated with B16 melanoma cells expressing OVA were immunized with SVF SPHs and/or amDC by sub‐renal capsule transplantation and monitored (**Figure** **6**a). SVF SPH or amDC transplantation enhanced anti‐tumor effects significantly when compared with the non‐treated control group, and the SVF+amDC‐transplanted group further suppressed tumor growth and significantly enhanced the overall survival rate when compared with the control groups (Figures 6b,c, and S10a, Supporting Information). To evaluate the local anti‐tumor response at the tumor site, SVF SPHs and/or amDCs were directly injected into the tumor (Figure 6d). Consistently, tumor progression in SVF SPH+amDC‐transplanted group was significantly suppressed when compared with those in the control groups (Figures 6e,f, and S10b, Supporting Information).

To assess the recruitment and retention of antigen‐specific T cells in the tumor, we analyzed tumor‐infiltrating lymphocytes after intratumoral injection of SVF SPHs and/or amDCs (Figure 6g,h). The frequencies of both OVA‐specific CD4^+^ and CD8^+^ CD44^+^ effector and CD44^+^ CD62L^+^ memory T cells normalized per gram of tumor weight in the SVF SPH+amDC‐transplanted group were significantly higher than those in control groups (Figure 6g). Moreover, confocal imaging revealed that injection with SVF SPHs and amDCs together induced greater T cell infiltration into the tumor than in control groups (Figure 6h).

The percentages of PD‐1^+^ OVA‐specific T cells in the tumor tissues were not significantly different among the experimental groups (Figure S10c, Supporting Information), and no significant improvements were observed in the combination therapy of SVF SPH+amDC intratumoral injection and anti‐PD‐1 therapy (Figure S11, Supporting Information). We confirmed tumor growth suppression mediated by SVF SPH+amDC immunization in an antigen‐dependent manner (Figure S12, Supporting Information). We observed the significant anti‐tumor effect only in the growth of B16 melanoma expressing OVA by the therapeutic regimen, but not in B16 melanoma without OVA expression. Overall, the data indicate that combined treatment with SVF SPHs and DCs enhanced the DC‐mediated anti‐tumor effect by facilitating T cell recruitment and formation of T cell lymphoid clusters in situ, thereby inducing significantly higher antigen‐specific T cell immunity than that of the exclusive DC therapy.

## 3 Discussion

**Figure 7:**
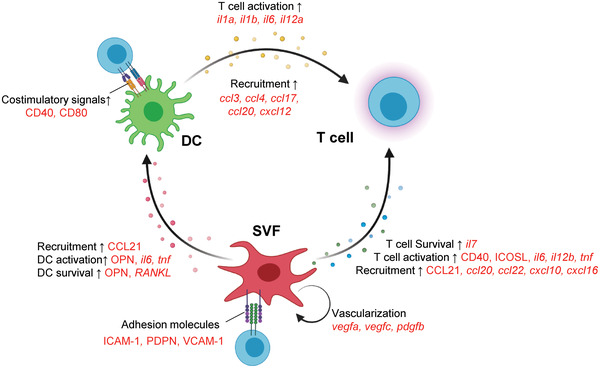
Proposed mechanism for intensified antigen‐specific T cell response and anti‐tumor effect by SVF SPHs and amDCs‐based immunotherapy. Italic, confirmed by mRNA expression; Capital, confirmed by protein expression.

The roles of TLS associated with favorable prognosis in cancer may include increasing cell‐to‐cell contact between antigen‐presenting cells and lymphocytes, thereby enhancing adaptive immune response specific to locally encountered neoantigens. The present study aimed to establish a niche for TLS formation that involves interplay between hematopoietic immune cells and non‐lymphoid stromal cells, based on communication through cytokines, chemokines, adhesion molecules, and survival factors. Here, we describe a novel approach using SVF cells and DCs for synergistic enhancement of DC‐mediated T cell response. The potential mechanism is outlined in **Figure** **7**. Expanded SVF cells and SVF spheroids augmented DC and T cell recruitment, activation, and survival through chemokines and cytokines, leading to increased production of costimulatory molecules and cytokines by DCs, which subsequently enhanced T cell activation. The expression of genes involved in T cell functional regulation, such as *nos2* and *cox2*, was also increased in SVF cells expanded with mDC coculture. These results demonstrate that expanded SVF cells could functionally resemble FRCs (Figure 2c).^[^ ^27^, ^28^, ^34^ ^]^ Moreover, DCs and SVF cells may work synergistically through chemokine secretion and enhance T cell infiltration to form in situ T cell clusters. Further, upregulation of adhesion molecules on SVFs can help them capture and retain DCs and T cells. TLS‐associated fibroblasts secrete proangiogenic factors.^[^ ^35^ ^]^ SVFs also promote tissue remodeling and vascularization^[^ ^36^, ^37^, ^38^ ^]^ via upregulation of genes, such as *vegfa, vegfc*, and *pdgfb*, when incubated with DCs (Figures 2g and S3g, Supporting Information), or cultured as spheroids (Figures 3 and S5b, Supporting Information). Indeed, vessel‐like structures generated by SVF SPHs in vitro provided a cavity for cell migration (Figure S13a–d, Supporting Information); blood vessels were also formed in vivo (Figure S13e, Supporting Information). The active generation of vasculature could further support DC and T cell trafficking, thereby enhancing systemic adaptive immune response specific to locally encountered antigens (Figure 7).

Immune cells and local stromal populations act as surrogate lymphoid tissue inducer cells (LTi) and lymphoid tissue organizer cells (LTo), respectively, at the initial phase of TLS formation.^[^ ^39^ ^]^ Our findings indicate that expanded SVF cells and NP‐OVA‐loaded mDCs may play roles as surrogate cells of LTo and LTi, respectively, to induce T cell clusters. Although the T cell lymphoid clusters induced by SVF SPHs and mDCs were not equipped with conventional lymphoid structures and failed to recruit B cells, they contributed to the anti‐cancer response by enhancing antigen‐specific adaptive immunity, especially CD4^+^ T cell immunity. Antigen‐specific CD4^+^ T cells within artificial TLSs could be cytotoxic in B16 melanoma and support anti‐tumor responses.^[^ ^40^, ^41^ ^]^ Given that effector T cells are initially recruited in the early phase of TLS formation,^[^ ^39^ ^]^ SVF SPH with mDCs transplantation efficiently summoned effector T cells into both renal capsule and tumor mass. Nonetheless, CD8^+^ T cells are undeniably major T cell subsets responsible for anti‐cancer immunity and should be strategically employed in further studies. Considering that recruitment of CD8^+^ T cells is accompanied by B cell recruitment in TLS, focusing future studies on developing B cell recruitment techniques would further enhance the functionality of TLS.^[^ ^21^ ^]^ Incorporation of TLR ligands that can induce B cell‐attracting chemokines, such as CXCL13, into the cellular transplants, is a potential therapeutic option (Figure S1h, Supporting Information). Overall, we demonstrate that SVFs can build an adequate environment to acquire and boost antigen‐specific immunity. SVF‐based cellular treatment can be further applied in combination with professional antigen‐presenting cells (DCs, B cells, and macrophages) as well as effector cells, such as CAR‐T cells, to provide a favorable immune microenvironment and promote immune cell recruitment, survival, and activation.

## 4 Conclusion

In summary, we demonstrated that expanded SVF cells from the adipose tissue showed molecular and functional similarities to lymph node stromal cells, especially FRCs. Coculturing SVF cells and mDCs augmented reciprocal enhancement of genes and molecules involved in T cell immunostimulation to one another, suggesting SVF cells provided a specialized niche to leverage DC‐mediated T cell responses. Assembly of SVF cell culture into 3D spheroids rendered further molecular and functional upregulation related to T cell chemotaxis and simulated in vivo T cell recruitment. Incorporation of SVF SPH and antigen‐loaded DCs efficiently elicited in vivo T cell migration into the engraftment sites and formed in situ ectopic T cell lymphoid clusters. Induction of these ectopic clusters showed both local and systemic antigen‐specific T cell responses against B16 melanoma and significantly controlled tumor growth compared to the controls. Here we demonstrated a novel strategy to employ SVF cells and DCs for synergistic enhancement of DC‐mediated immunotherapy by inducing the formation of in situ ectopic T cell clusters and enhancing T cell recruitment and responses in an antigen‐specific manner.

## 5 Experimental Section

### Mice

C57BL/6 wildtype mice (Koatech, Seoul, Republic of Korea) were used for ex vivo isolation of SVF cells. C57BL/10NAGCSnAi‐(KO) Rag2 (H‐2b) mice (Taconic Biosciences, NY, USA) were used for the isolation of DCs. C57BL/6‐Tg(TcraTcrb)1100Mjb/J and B6.Cg‐Tg(TcraTcrb)425Cbn/J mice (The Jackson Laboratory, ME, USA) were used as positive controls in OVA‐specific immune response analysis. All mice were housed and maintained in a pathogen‐free facility at Seoul National University (SNU) College of Medicine. All mouse experiments were approved by the SNU Institutional Animal Care and Use Committee (permission ID: SNU‐150115‐2‐7, SNU‐160212‐2‐7, SNU‐171123‐4‐7). To isolate SVF cells from mice with abundant adipose tissue, all C57BL/6 mice used were male, and other mouse strains used were matched male as well. All mice were used at 6–12 weeks of age, with a 20–25 g average weight.

### Expansion of Stromal Cells and SVF Spheroid Culture

To extract SVF cells and FRCs, visceral and skin fat tissues and inguinal lymph nodes were collected and finely chopped (Figure S1a, Supporting Information). The tissues were incubated with 0.8 mg ml^−1^ Collagenase IV (Gibco, MA, USA) and 100 µg ml^−1^ DNaseI (Worthington, OH, USA) in sterile Hank's balanced salt solution (Intron, Seoul, Republic of Korea), including 3% bovine serum albumin (BSA) (MP biomedicals, CA, USA), 1.0 mM CaCl_2_ (Sigma‐Aldrich, MO, USA), and 0.8 mM MgCl_2_ (Sigma‐Aldrich) for 1 h at 37 °C on a shaker at 170 rpm. After centrifugation at 500 ×*g* for 3 min, the supernatant was discarded, and the pellet was resuspended and incubated in Dulbecco's modified Eagle's Medium (Welgene, Daegu, Republic of Korea) containing 10% fetal bovine serum (Gibco) and 1% penicillin/streptomycin (Gibco). For generating SVF SPHs, SVF cells cultured for 6 to 10 d were seeded in a spheroid film (Incyto, Cheonan, Republic of Korea) and incubated for 2 d. One spheroid film generated 361 (19 × 19) SVF SPHs.

### Preparation of Fe_3_O_4_–ZnO Core‐Shell NPs

Fe_3_O_4_–ZnO core‐shell NPs with sizes of 10.5 ± 1.5 nm were synthesized via a previously reported nanoemulsion method with slight size modification for the current study.^[^ ^24^, ^42^ ^]^ The thickness of the ZnO shell to reduce its cytotoxicity was decreased. First, for the synthesis of the Fe_3_O_4_ core, 0.1766 g of iron (III) acetylacetonate (0.5 mmol), 0.6468 g of 1,2‐hexadecandiol (2.5 mmol), and 0.7530 g of poly(ethylene glycol)‐block‐poly(propylene glycol)‐block‐poly(ethylene glycol) (Sigma‐Aldrich) were dissolved in 15 mL of octyl ether 25 °C. The mixture was gradually heated to 120 °C in a vacuum and then rapidly heated to 300 °C for refluxing in an argon (Ar) atmosphere. After 1 h of refluxing, the black‐colored solution was slowly cooled to 25 °C. To form the ZnO shell, 0.2636 g of zinc acetylacetonate (1.0 mmol), 0.6468 g of 1,2‐hexadecanediol (2.5 mmol), and 5 mL of octyl ether were added to the solution containing the Fe_3_O_4_ core. The mixed solution was heated to 80 °C at a constant heating rate of 1°C min^−1^ and homogenized at 80 °C for 2 h in an Ar atmosphere. After refluxing the solution for 1 h at 300 °C, the products were precipitated by ethanol and washed at least five times using ultrasonication and centrifugation.

### Characterization of Fe_3_O_4_–ZnO Core‐Shell NPs

The morphology and microstructure of the Fe_3_O_4_–ZnO core‐shell NPs were analyzed using transmission electron microscopy (TEM) (FEI, Tecnai F20 G2) at an accelerating voltage of 200 kV. Detailed dark‐field and elemental‐mapping images were obtained using analytical TEM (FEI, Talos F200X) at 200 kV. The samples for the TEM experiments were prepared by washing them in absolute ethanol and then dropped onto the TEM grid. The sizes of the samples, which followed a Gaussian distribution, were measured from their TEM images. X‐ray photoelectron spectroscopy (Thermo Scientific, K‐Alpha^+^) data were collected using a monochromated Al *Kα* X‐ray source operating at 1486.6 eV. A survey scan including the photoemission features from all elements and narrow scans of detailed elemental regions (Fe 2*p*, Zn 2*p*, and O 1*s*) were collected with pass energies of 200 eV at 1.0 eV intervals and 50 eV at 0.1 eV intervals, respectively. All energy‐loss regions in the narrow scan spectra were fitted using Shirley background functions.

### Production of Recombinant ZBP‐OVA Proteins

The genomic DNA of the B16MO5 cell line, which expresses OVA, was extracted using DNeasy Blood & Tissue Kits (Qiagen, Hilden, Germany) according to the manufacturer's instructions. Extracted DNA was PCR‐amplified (forward primer (FP): 5′‐TTT GAA TTC ATG GGC TCC ATC GGT GCA‐3′ and reverse primer (RP): 5′‐AAA CTC GAG AGG GGA AAC ACA TCT GCC‐3′; *Eco* RI and *Xho* I restriction sites underlined) and cloned into pET23d‐ZBP vector encoding 3xZBP (*Bam* HI‐RPHRKGGDARPHRKGGDARPHRKGGDA‐*Eco* RI).^[^ ^24^ ^]^ *Escherichia coli*, HIT Competent BL21 strain (RBCBioscience, New Taipei City, Taiwan), was transformed into the cloned vector pET23d‐ZBPOVA, and ZBPOVA was then purified using a protocol adapted from previously described methods.^[^ ^43^ ^]^ Endotoxin was removed from the purified protein using Triton X‐114 (Sigma‐Aldrich). Endotoxin contamination of the purified protein was determined using a Pierce LAL Chromogenic Endotoxin Quantification Kit (Thermo Fisher Scientific, MA, USA) according to the manufacturer's instructions. The purified ZBPOVA was quantified using sodium dodecyl sulfate‐polyacrylamide gel electrophoresis and stored at −80 °C until further use.

### DC Isolation, Antigen Delivery, and Maturation

Bone marrow of C57BL/10NAGCSnAi‐(KO) Rag2 (H‐2b) was isolated and differentiated into DCs as previously described.^[^ ^24^ ^]^ Briefly, isolated bone marrows were incubated in Iscove's modified Eagle's medium (IMDM) (Gibco) supplemented with 10% fetal bovine serum, 1.5 ng mL^−1^ recombinant mouse GM‐CSF (PeproTech, NJ, USA), 1.5 ng mL^−1^ mouse interleukin (IL)‐4 (PeproTech), 1% penicillin/streptomycin, 50 µg ml^−1^ gentamicin (Gibco, MA, USA), 2 mM l‐glutamine (Gibco), and 50 nM *β*‐mercaptoethanol (Gibco). The medium was replaced every other day for 6–8 days, and immature DCs were collected for subsequent experiments. For intracellular antigen delivery to immature DCs, Fe_3_O_4_–ZnO core‐shell NPs were used. NPs (50 to 100 µg) were washed with phosphate‐buffered saline three times and incubated with 20 µg ZBP‐OVA for 1 h at 25°C to form NP‐OVA. NP‐OVA was washed with IMDM three times and then incubated with 1 × 10^6^ immature DCs for 1 h at 37 °C. To maturate DCs, DCs were treated with 1 mg ml^−1^ lipopolysaccharides for 16–18 h and washed with IMDM three times before use.

### Flow Cytometric Analysis and Antibodies

Cells were blocked with super‐block solution containing 10% goat serum (Thermo Fisher Scientific), 10% rat serum (Thermo Fisher Scientific), 10% mouse serum (Sigma‐Aldrich), and 10 µg/ml of anti‐CD16/CD32 (2.4G2) antibody (BD Pharmingen, NJ, USA), followed by surface staining for 30 min on ice with APC/Cy7‐conjugated anti‐CD45, BV605‐conjugated anti‐CD11c, Alexa488‐conjugated anti‐CD11c, APC‐conjugated anti‐B220, PE‐conjugated anti‐CD11b, APC‐conjugated anti‐PDPN, FITC‐conjugated anti‐ ICAM‐1, PE/Cy7‐conjugated anti‐CD31, PE‐conjugated anti‐LT*β*R, FITC‐conjugated anti‐VCAM‐1, PE‐conjugated anti‐CD140*α*, PE‐conjugated anti‐CD34, PerCP/Cy5.5‐conjugated anti‐CD44, PE/Cy7‐conjugated anti‐CD86 (Biolegend, CA, USA), PE‐conjugated anti‐CD3, BV421‐conjugated anti‐CD3, PerCP‐conjugated anti‐CD4, FITC‐conjugated anti‐CD8, APC‐conjugated anti‐Gr‐1, BV605‐conjugated anti‐CD62L, FITC‐conjugated anti‐I‐Ab and PE‐conjugated anti‐CD40, PE‐conjugated anti‐Sca‐1 (Ly6A/E) (BD Pharmingen), PE/Cy7‐conjugated anti‐CD4, eFlour 450‐conjugated anti‐CD3, PE/Cy7‐conjugated anti‐F4/80, and APC‐conjugated anti‐CD80 (eBioscience, CA, USA). Live cells were identified by staining with Zombi Aqua (Biolegend, CA, USA). The cells were quantified using LSRFortessa X‐20 flow cytometer, BD LSRII flow cytometer (BD Pharmingen), and CytoFLEX S (Beckman Coulter, CA, USA). Data were analyzed using FlowJo software (Tree Star, Ashland, OR, USA).

### Sub‐Renal Capsule Transplantation

Transplantation underneath the kidney capsule was performed as previously described.^[^ ^44^ ^]^ In brief, the mouse was anesthetized with isoflurane inhalation, its flank was shaved and disinfected, and a small incision was made in both skin and peritoneum to externalize the kidney out of the abdominal cavity. The surface capsule on the kidney was slightly scratched using a 30‐gauge needle (BD Pharmingen) to allow the graft to be inserted using a threaded plunger (Hamilton, NV, USA). Once all were inserted, the graft site was dried and cauterized (Bovie medical corporation, NY, USA). The kidney was then carefully placed back into the cavity, and the incisions were sutured.

### qPCR

To confirm the mRNA expression of the principal chemokines of LN stromal cells in SVF cells, RNA extraction was performed using Trizol reagent (Invitrogen, CA, USA) following standard protocols. Subsequently, complementary DNA was synthesized using a reverse transcript premix kit (Intron). qPCR was carried out using the SYBR Green master mix (Life Technologies, CA, USA) and a CFX Real‐Time PCR Detection System (Bio‐Rad Laboratories, CA, USA). Each sample was examined in duplicate. Relative mRNA expression was calculated based on the values of *β‐actin* (*Actb*) (Primers, *Ccl19* FP: 5′‐CCT GGG AAC ATC GTG AAA GC‐3′ and RP: 5′‐TAG TGT GGT GAA CAC AAC AGC‐3′, 81 base pair, *Ccl21* FP: 5′‐ GTG ATG GAG GGG GTC AGG A ‐3′ and RP: 5′‐ GGG ATG GGA CAG CCT AAA CT ‐3′, 109 base pair, *Cxcl13* FP: 5′‐ GGCCACGGTATTCTGGAAGC ‐3′ and RP: 5′‐ GGGCGTAACTTGAATCCGATCTA ‐3′, 108 base pair, *Cxcl12* FP: 5′‐ CAT CAG TGA CGG TAA ACC AG ‐3′ and RP: 5′‐ CAC AGT TTG GAG TGT TGA GG ‐3′, 116 base pair, *Actb* FP: 5′‐ TGT TAC CAA CTG GGA CGA CAT G ‐3′ and RP: 5′‐ GGG GTG TTG AAG GTC TCA AAC ‐3′, 165 base pair).

### Coculturing SVF Cells and DCs

SVF cells (2 × 10^4^ per well) expanded with mDCs (1 × 10^6^ per well) were cultured in 24‐well plates for 24 h. Subsequently, they were separated by magnetic cell separation (MACS; Miltenyi Biotec, Bergisch Gladbach, Germany) using anti‐CD45 microbeads for mRNA sequencing of each cellular subset.

### mRNA Sequencing and Analyses

The quality of the RNA isolated from SVF cells, SVF SPHs, and DCs was measured using an Agilent 2100 bioanalyzer with the RNA 6000 Nano Chip (Agilent Technologies, Amstelveen, Netherlands), and the RNA was quantified using an ND‐2000 Spectrophotometer (Thermo Fisher Scientific). Libraries were generated using a QuantSeq 3’ mRNA‐Seq Library prep kit (Lexogen, Inc., Austria) (Figure 2) or a SMARTer Stranded RNA‐Seq Kit (Clontech Laboratories, CA, USA) (Figure 3). Polyadenylated mRNA was specifically isolated using a Poly(A) RNA Selection Kit (LEXOGEN, Vienna, Austria), followed by complementary DNA synthesis, shearing for fragmentation, and amplification using PCR. Agilent 2100 bioanalyzer was used to measure the mean fragment size, and quantification was performed using a StepOne Real‐Time PCR System (Life Technologies). The libraries were sequenced on single‐end 75 sequencing using NextSeq 500 (Illumina, CA, USA) (Figure 2) or paired‐end 100‐bp reads on HiSeq 2500 (Illumina, CA, USA) (Figure 3). The sequenced reads were mapped using TopHat. Data were analyzed using Metascape based on Gene Ontology (GO) terms,^[^ ^45^ ^]^ ExDEGA (E‐biogen, Seoul, Republic of Korea), and visualized using Graphpad Prism 8 (GraphPad Software Inc., San Diego, CA, USA). To assess the functional similarities between expanded SVF cells and FRCs, gene expression datasets from a recent mRNA‐seq study (GSE147357) were retrieved.^[^ ^29^ ^]^ CPM (counts per million mapped reads) was calculated using the Quantseq data and integrated with RPKM (reads per kilobase of transcript per million mapped reads) from GSE147357 as Quantseq generated only one fragment per transcript. Batch effects between two datasets were removed using the “removeBatchEffect” function in the limma R package (version 3.46.0). PCA was performed using ‘prcomp’ function in R v4.0.5 (R Foundation for Statistical Computing, Vienna, Austria).

### Enzyme‐Linked Immunosorbent Assay (ELISA)

The expression of CCL21 and osteopontin in cell culture supernatants was measured using ELISA kits purchased from Research and Diagnostic Systems (MN, USA) and Lifespan Biosciences (WA, USA), respectively, according to the manufacturer's instructions.

### Real‐Time Chemotaxis Assay

To confirm the chemoattractant ability of SVF SPHs, a µ‐Slide Chemotaxis kit (Ibidi, Gräfelfing, Germany) was used according to the manufacturer's protocol. SVF SPHs or control media were injected into the left chamber, and splenocytes were inserted into the right chamber. Type I Collagen solution (1.5 mg ml^−1^; Corning, NY, USA) was injected into the observation area in between two chambers. The slide was incubated for 24 h to track cells and analyze their migration.

### In Vitro and In Vivo Imaging

SVF cells and SVF SPHs in the cell culture plate were observed under a microscope (Olympus Corporation) using VisiView (Visitron Systems, Puchheim, Germany). To demonstrate the chemoattractant ability of SVF SPHs, live video imaging was performed during 24 h of incubation of the slide using FV1000 (Olympus Corporation, Tokyo, Japan). To analyze the results of in vivo transplantation, the grafts were collected two weeks after the operation and embedded with frozen section media (Leica Biosystems, IL, USA), and rapidly frozen in liquid nitrogen. The samples were cryo‐sectioned to a thickness of 5–6 µm and stored at ‐80°C until further experiments. For histological analysis, the cryo‐sectioned segments were stained with hematoxylin and eosin. For confocal imaging, the sectioned samples were fixed in 4% paraformaldehyde and stained with FITC‐labeled anti‐CD3, FITC‐labeled anti‐CD8 (BD Pharmingen), Alexa647‐labeled anti‐CD31, Alexa594‐labeled anti‐PDPN, Alexa647‐labeled anti‐CD11c, Alexa 647‐labeled anti‐CD4 (Biolegend, CA, USA), Alexa 594‐labeled anti‐VEGFR3 (Bioss Antibodies, MA, USA), and 4′,6‐diamidino‐2‐phenylindole (DAPI; Thermo Fisher Scientific). Confocal images were taken using FV3000 (Olympus Corporation) and analyzed using IMARIS v9.3 (Bitplane, Zürich, Switzerland)

### Assessment of Antigen‐Specific Immune Response

To determine whether SVF SPHs plus DCs can effectively induce an antigen‐specific immune response, the combinations (1 × 10^6^ DCs per mouse and/or 361 SVF SPHs/mouse) were transplanted underneath the kidney capsule twice with a two‐week interval. DCs were pulsed with NP‐OVA, and the same number of NP‐OVA was included in the SVF SPH‐transplanted group. After immunizing the C57BL/6 mice, their splenocytes were stained with anti‐MHC tetramers for 30 min at 4 °C, followed by surface marker staining for an additional 30 min. APC‐labeled tetramer SIINFEKL‐H‐2Kb and PE‐labeled tetramer AAHAEINEA‐I‐Ab were kindly provided by the National Institutes of Health Tetramer Core Facility in the US.

### Measurement of Anti‐Tumor Effect

OVA‐expressing melanoma B16MO5 cells or control B16F10 cells were inoculated to the left flank of mice as indicated. To measure the systemic effect, the combinations with 1 × 10^6^ DCs per mouse and/or 361 SVF SPHs per mouse were engrafted via sub‐renal capsule transplantation three times at weekly intervals (Figure 6a). For combination therapy with anti‐PD‐1 therapy, mice were inoculated with B16MO5 cells and systematically immunized with sub‐renal capsule transplantation of SVF SPH and amDCs and intraperitoneally injected with 100 µg of anti‐PD‐1 antibody or IgG isotype three times, at weekly intervals (Figure S11, Supporting Information) For the local anti‐tumor effect, the combinations were directly injected into the tumor once, seven days after tumor inoculation. The tumor volume (mm^3^) was calculated as 1/2 × [(short diameter) × (long diameter)^2^]. The experiment was terminated when the tumor size measured was > 2000 mm^3^.

### Statistical Analysis

Data were analyzed using GraphPad Prism 8 (Graphpad Software Inc.) for statistical analysis. RNA‐seq analysis was performed using ExDEGA v 2.5 (E‐biogen).

## Conflict of Interest

The authors declare no conflict of interest.

## Author Contributions

J.W.L., B.C.P., and N.Y.J. contributed equally to this work. J.W.L designed and performed the majority of the experiments. B.C.P and S.W.B synthesized and characterized the nanoparticles. J.W.L. and N.Y.J. with the assistance of S.L, U.P., H.J.R., and H.R.P. performed transplantation experiments into kidney capsules. J.W.L., N.Y.J., S.L, and Y.K. with the assistance of Y.H.J performed tumor‐infiltrating lymphocyte isolation, and D.S.L confirmed the data. J.W.L with the assistance of P.S. optimized SVF cell isolation. Y.K.C performed 3D imaging, and S.C. confirmed the data. J.W.L and K.J analyzed transcriptomic data. J.W.L., B.C.P., N.Y.J., Y.K.K., and N.H.C. confirmed the data and wrote the manuscript. Y.K.K. and N.H.C. supervised the entire project. All authors contributed to the article and approved the submitted version.

## Supporting information

Supporting Information

Supplemental Movie 1

Supplemental Movie 2

## Data Availability

The data that support the findings of this study are available from the corresponding author upon reasonable request.
