# Supplementary material for: Inducing Ectopic T Cell Clusters Using Stromal Vascular Fraction Spheroid‐Based Immunotherapy to Enhance Anti‐Tumor Immunity
Source: Adv Sci (Weinh). 2022 Sep 4;9(28):2203842. doi: 10.1002/advs.202203842 (PMC9534947; doi:10.1002/advs.202203842)
Supplement: Supplementary file 1 — Supporting Information [file ADVS-9-2203842-s003.pdf]

ADVANCED SCIENCE
Open Access

Supporting Information

for *Adv. Sci.*, DOI 10.1002/advs.202203842

Inducing Ectopic T Cell Clusters Using Stromal Vascular Fraction Spheroid-Based Immunotherapy to Enhance Anti-Tumor Immunity

Jae-Won Lee, Bum Chul Park, Na Yoon Jang, Sihyeon Lee, Young Kyu Cho, Prashant Sharma, Sang Won Byun, Kyeongseok Jeon, Yun-Hui Jeon, Uni Park, Hyo Jin Ro, Hyo Ree Park, Yuri Kim, Dong-Sup Lee, Seok Chung, Young Keun Kim and Nam-Hyuk Cho**

Supporting Information

Inducing ectopic T cell clusters using stromal vascular fraction spheroid-based immunotherapy to enhance anti-tumor immunity

Jae-Won Lee, Bum Chul Park, Na Yoon Jang, Sihyeon Lee, Young Kyu Cho, Prashant Sharma, Sang Won Byun, Kyeongseok Jeon, Yun-Hui Jeon, Uni Park, Hyo Jin Ro, Hyo Ree Park, Yuri Kim, Dong-Sup Lee, Seok Chung, Young Keun Kim, and Nam-Hyuk Cho**

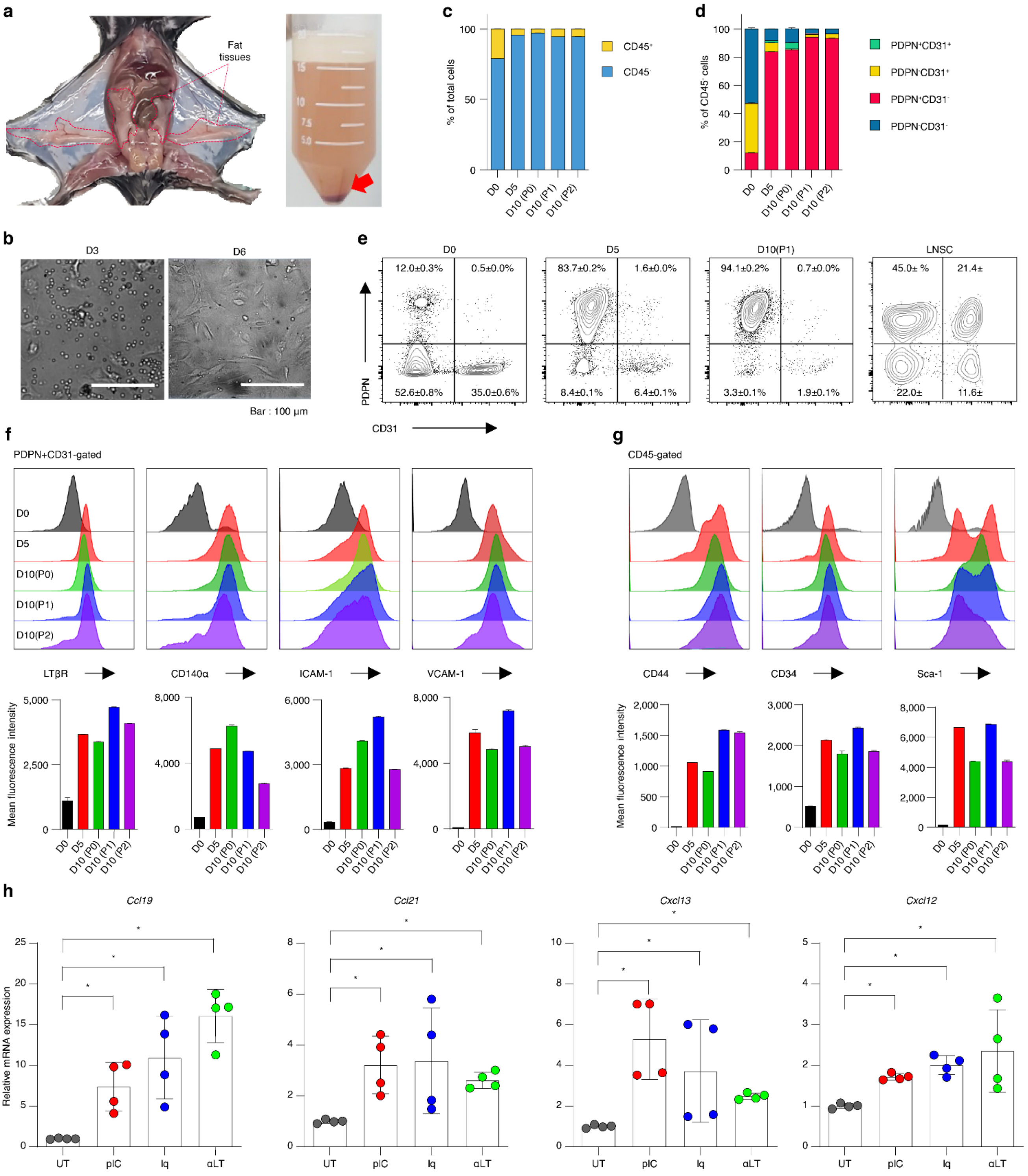

Figure S1. Characteristics of expanded stromal vascular fraction (SVF) cells. a) Isolation of SVF cells from adipose tissues. Skin and visceral fat tissues were used to extract SVF cells (left, red dotted line). After digestion and centrifugation, pelleted SVF cells were distinguished from

adipocytes. (right, red arrow). b) Microscopic images of SVF cells cultured in complete medium for 3 or 6 d. Scale bar: 100 μm. c-g) Data from flow cytometric analysis of SVF cells cultured for indicated dates (D0, D5, and D10) and passaging (P0, P1, and P2 on D10) (*n* = 3, independent experiments per group). c) Frequency of $CD45^{-}$ cells and $CD45^{+}$ cells within the expanded SVFs. d) Ratio of cell populations express stromal cell subset markers, PDPN and CD31, within CD45- SVF cells cultured for indicated dates. e) Representative dot plots of the cell populations in (d) and lymph node stromal cells (LNSC) as positive control. f) Expression of FRC markers, LTβR, CD140α, ICAM-1 and VCAM-1, on SVF cells gated on $PDPN^{+}$ $CD31^{-}$ were displayed with representative histograms (top) and mean fluorescence intensity (bottom) of cells in indicated groups.). g) Expression of mesenchymal stem cell/stromal cell markers, CD44, CD34 and Sca-1, on SVF cells gated on CD45-. h) Relative mRNA expression of chemokines such as *Ccl19*, *Ccl21*, *Cxcl13* and *Cxcl12* in untreated group (UT), polyI:C- treated (pIC), imiquimod-treated (Iq) and anti-LTβR-treated group (αLT) (*n* = 4, mean ± s.e.m.). Each group was compared with UT. * $P < 0.05$ by two-tailed students' *t* test (h); PDPN, podoplanin, FRC, fibroblastic reticular cell; LTβR, lymphotoxin β receptor; ICAM-1, intracellular adhesion molecule-1; VCAM-1, vascular cell adhesion molecule-1.

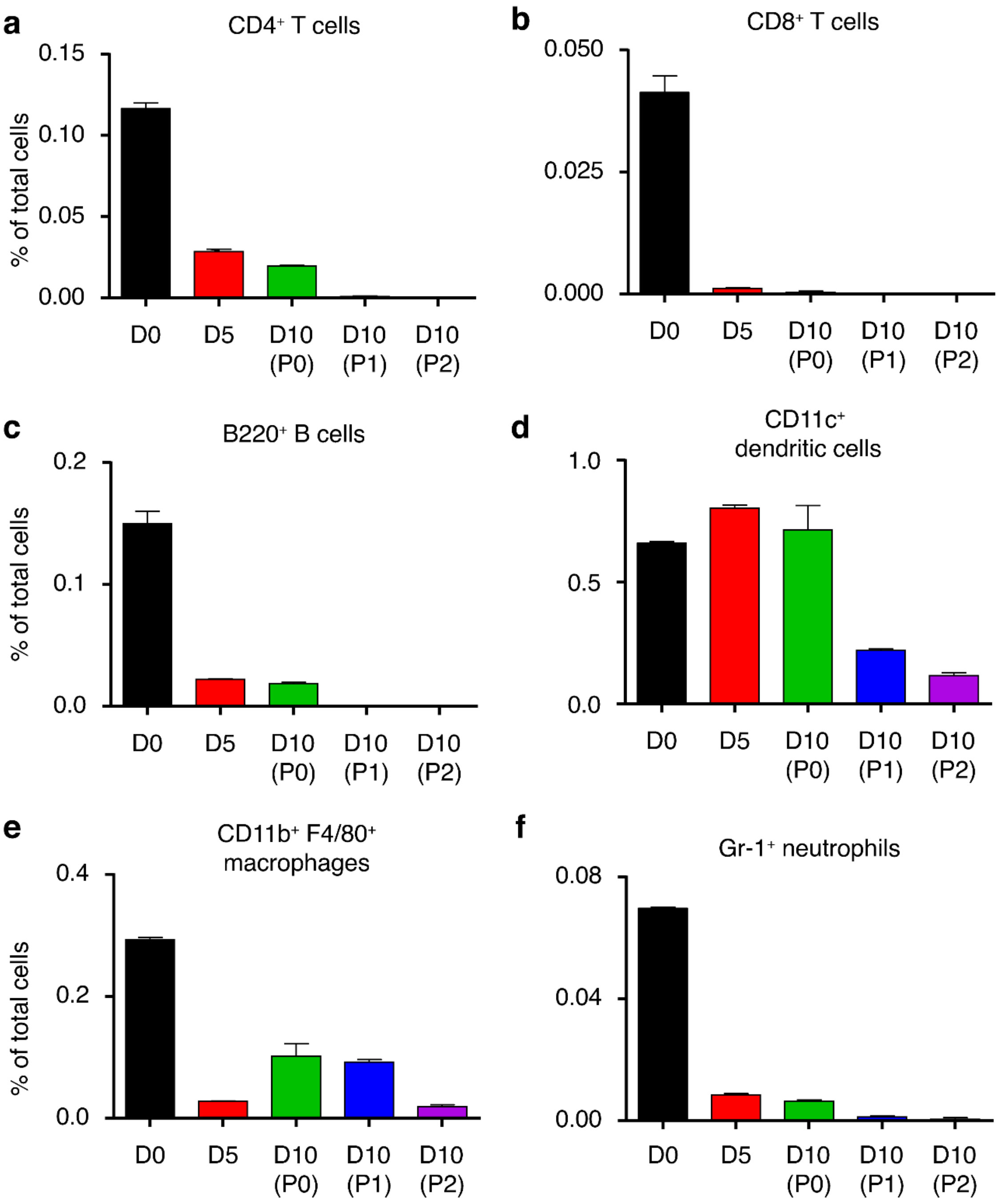

Figure S2. Frequency of immune cells in CD45^{+} hematopoietic cells in expanded SVFs. a-f) Frequency of CD4^{+} T cells a) CD8^{+} T cells b) B cells c) dendritic cells d) macrophages e) neutrophils f) in SVF cells cultured for indicated dates (D0, D5, and D10) and passaging (P0, P1, and P2 on D10) (*n* = 3, mean ± s.e.m., independent experiments per group).

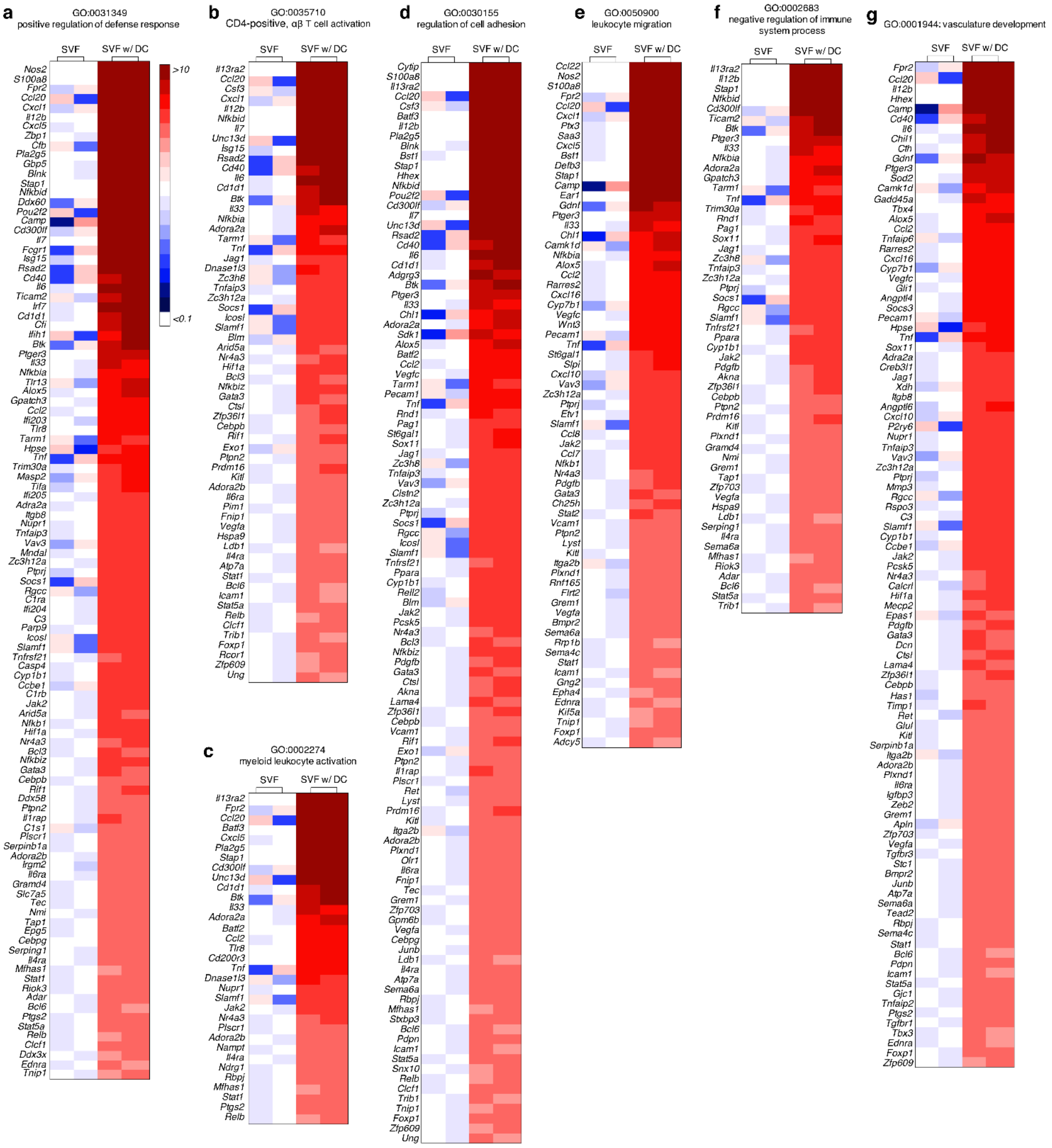

Figure S3. Heat map of genes involved in the selected enriched GO terms in SVF cells co-cultured with mDCs. a-g) Genes involved in the selected gene sets out of the top 20 upregulated gene sets in Figure 2b were illustrated using heat map: genes involved in positive regulation of defense response (GO:0031349). a) CD4-positive, αβ T cell activation (GO:0035710) b) myeloid leukocyte activation (GO:0002274) c) regulation of cell adhesion (GO:0030155) d) leukocyte

migration (GO:0050900) e) negative regulation of immune system process (GO:0002683) f) and vasculature development (GO:0001944) g). Cutoff by $p < 0.05$; GO, gene ontology; SVF) stromal vascular fraction; mDC, mature dendritic cells.

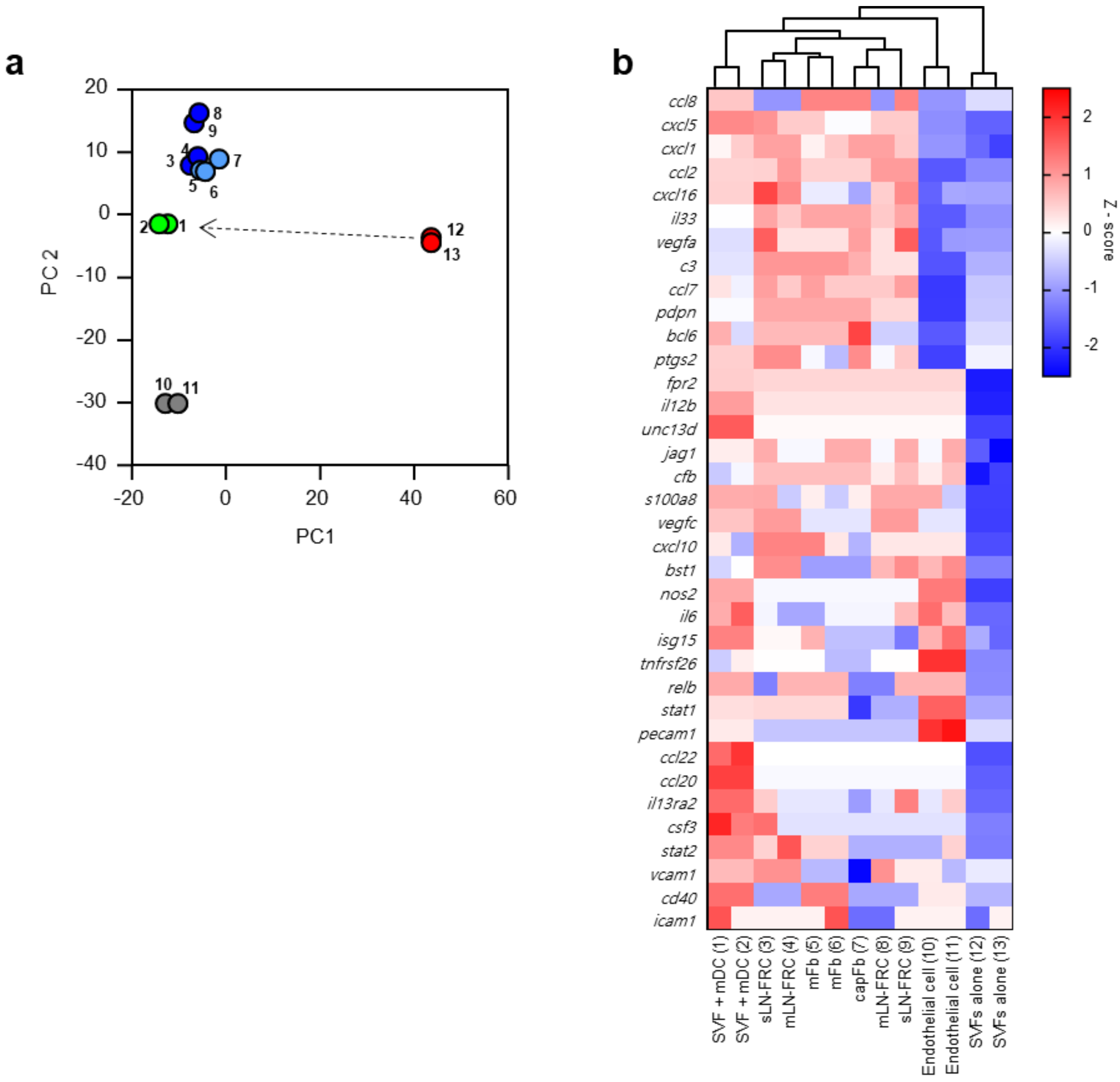

Figure S4. Comparison of the expression of differentially expressed genes (DEGs) between SVF cells co-cultured with mDCs and FRCs. The expression of the DEGs selected in Figure 2c was compared with those in datasets retrieved from recent mRNA-seq studies (ref 29). Numbers indicate individual samples. a) Principal Component Analysis (PCA) of SVF co-cultures with mature DCs and other lymphoid organ stromal cells. Dashed arrow indicates transcriptional shift of SVFs by co-culture with mDCs. b) Hierarchical clustering analysis of all samples (as in a) showing expression of

the genes that account for the differences in PC1 (n = 1 or 2). SVF) stromal vascular fraction; mDC, mature dendritic cells; FRC, fibroblastic reticular cell; sLN: skin-draining lymph node; mLN: mesenteric lymph node; mFb: thymic medullary fibroblast; capFb: thymic capsular fibroblast.

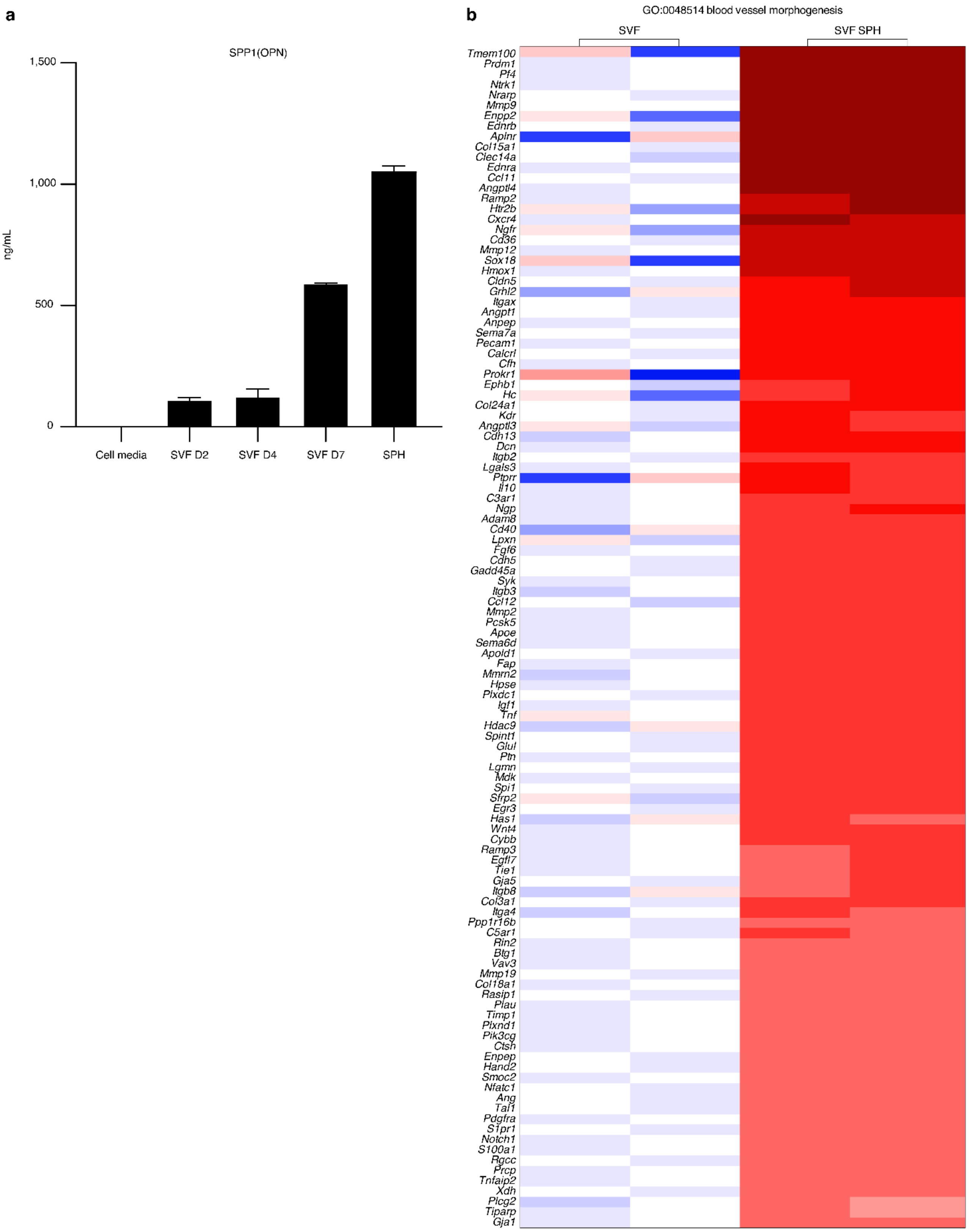

Figure S5. Characteristics of SVF SPH. a) Comparison of osteopontin expression (Spp1, OPN) in cell culture supernatants for indicated dates (D2, D4 and D7) using ELISA. SVF cells cultured for 6

d and additional 2 d for spheroid formation were used without media change (SPH). ($n = 3$, mean ± s.e.m.) b) Heat map of genes involved in blood vessel morphogenesis (GO:0048514), one of the top 20 upregulated gene sets in SVF SPH compared with those in 2D-cultured SVF. Cutoff at $p < 0.05$; SVF SPH, stromal vascular fraction cell spheroids; ELISA, enzyme-linked immunosorbent assay

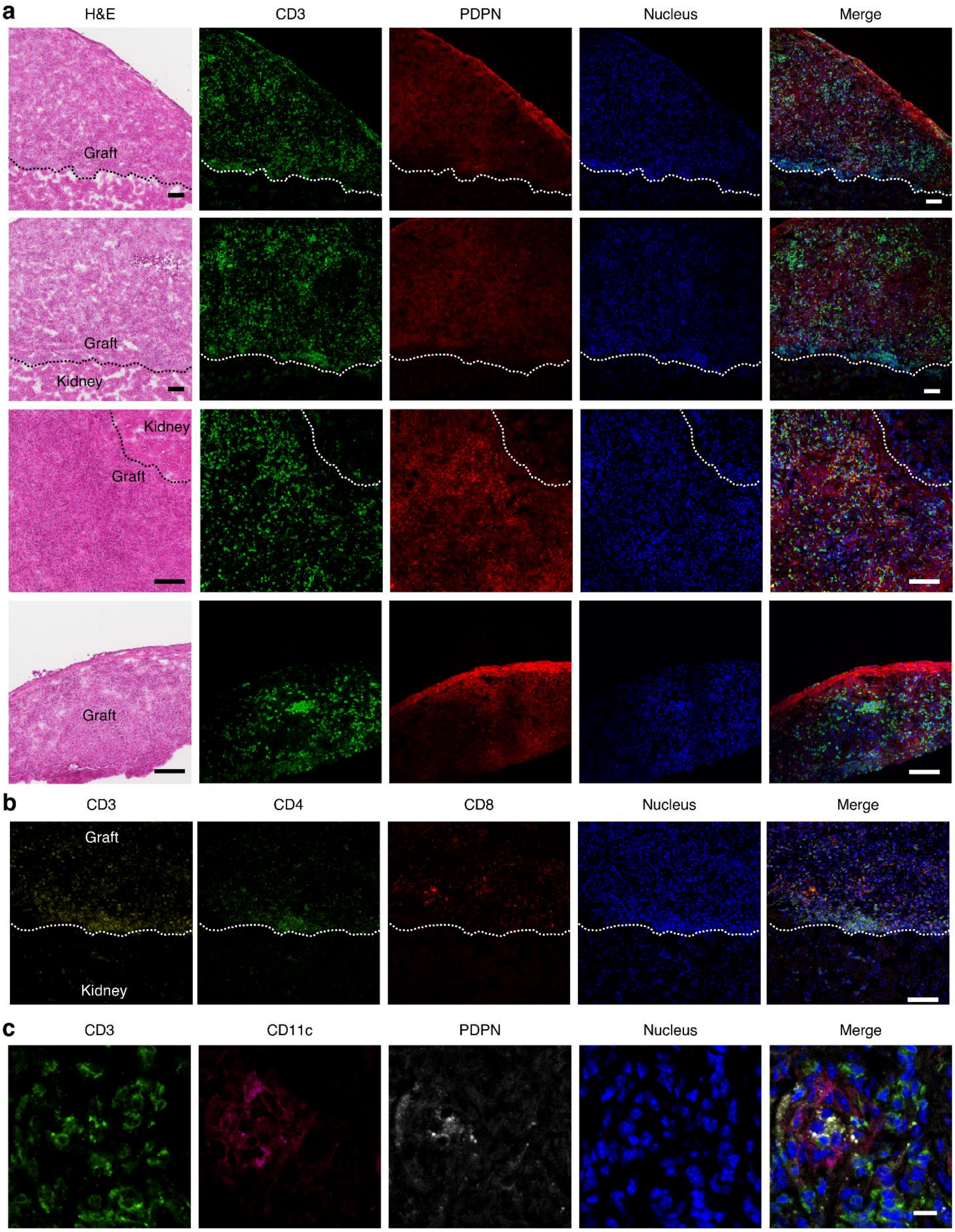
a
H&E
CD3
PDPN
Nucleus
Merge
Graft
Graft
Kidney
Kidney
Graft
Graft
b
CD3
CD4
CD8
Nucleus
Merge
Graft
Kidney
c
CD3
CD11c
PDPN
Nucleus
Merge

Figure S6. Confocal images of frozen sections of the grafts in SVF SPH + mDC-transplanted group. a) Tertiary lymphoid structures (TLS) consisting of T cells and DCs were observed in the graft underneath the kidney capsule. $CD3^+$ T cells were densely recruited, surrounded by $PDPN^+$ expanded SVF cells at the graft sites. Dotted lines: boundary lines between the graft and the kidney tissue. Scale bar: 100 μm. b) $CD4^+$ and $CD8^+$ T cells in confocal images. b) Enlarged images of $CD11c^+$ DCs and $CD3^+$ T cells in Figure 4d. Scale Bar: 10 μm; SVF SPH, stromal vascular fraction cell spheroids; mDC, mature dendritic cells; PDPN, podoplanin.

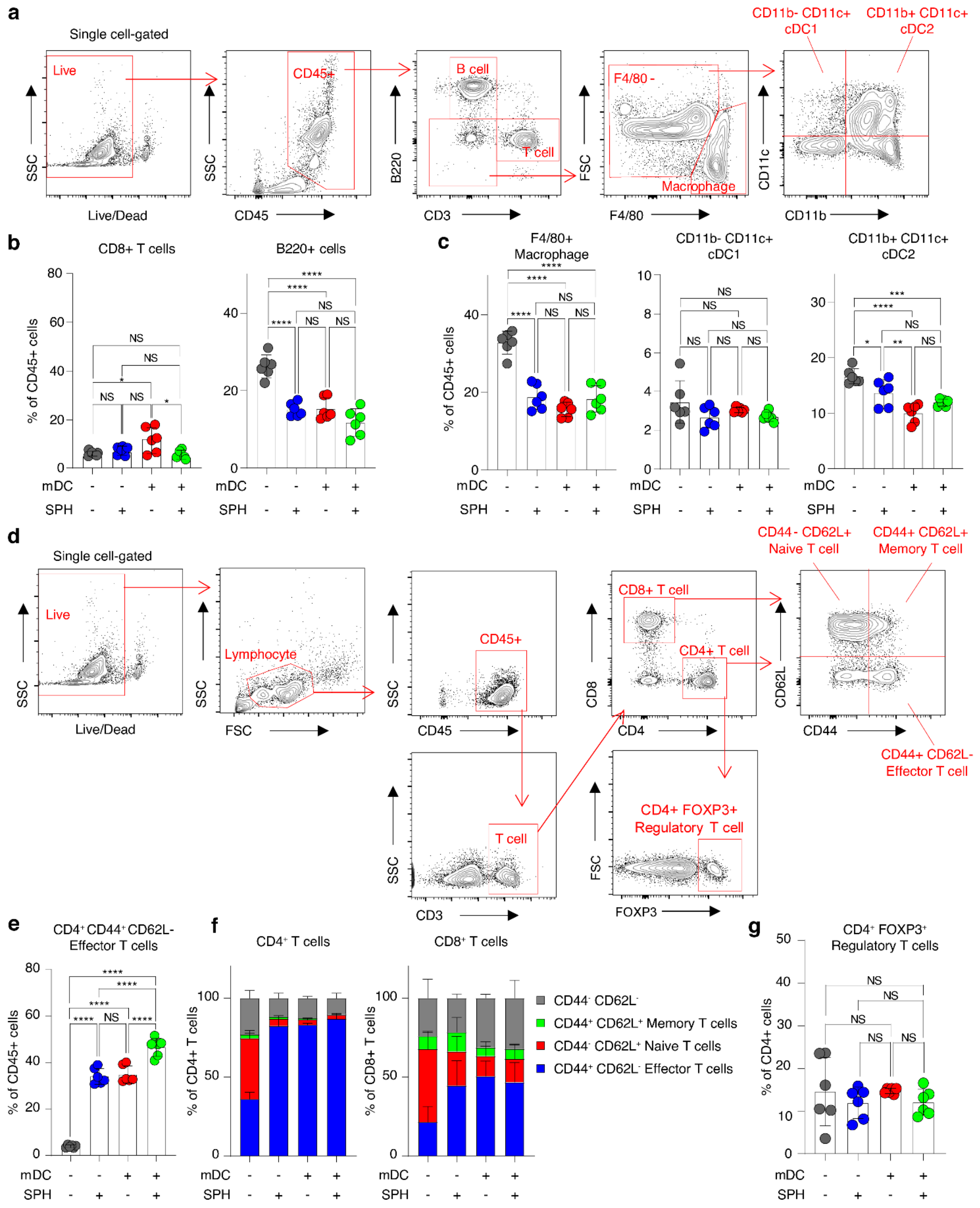

Figure S7. Identification of cellular components in the grafts transplanted underneath the

kidney capsule. a-c) Flow cytometric analysis for lymphoid and myeloid cells in untreated, SVF SPH (SPH)-, mDC-, SVF SPH + mDC-transplanted group (n = 6, mean ± s.e.m.). a) Representative dot plots of the gating strategy used. Frequency of $CD8^+$ T cells and B cells (b) and $F4/80^+$ macrophages, $CD11b^-$ $CD11c^+$ cDC1 and $CD11b^+$ $CD11c^+$ cDC2 (c). d-g) Flow cytometric analysis for T cell subsets such as naïve, effector, memory, and regulatory T cells in untreated, SPH-, mDC-, SVF SPH + mDC-transplanted group (n = 5, mean ± s.e.m.). d) Representative dot plots of the gating strategy used. e) Frequency of $CD4^+$ $CD44^+$ $CD62^-$ effector T cells in the indicated groups. f) Proportion of double negative, $CD44^+$ $CD62L^+$ memory, $CD44^-$ $CD62L^+$ naïve, $CD44^+$ $CD62L^-$ effector cells in $CD4^+$ and $CD8^+$ T cells. g) Frequency of $Foxp3^+$ regulatory T cells in $CD4^+$ T cells. *: $p < 0.05$, **: $p < 0.01$, ***: $p < 0.001$, NS, non-significant by one-way ANOVA with Tukey's post-test (b and c, e-g); SVF SPH, stromal vascular fraction cell spheroids; mDC, mature dendritic cells; cDC1, conventional type 1 dendritic cells; cDC2, conventional type 2 dendritic cells.

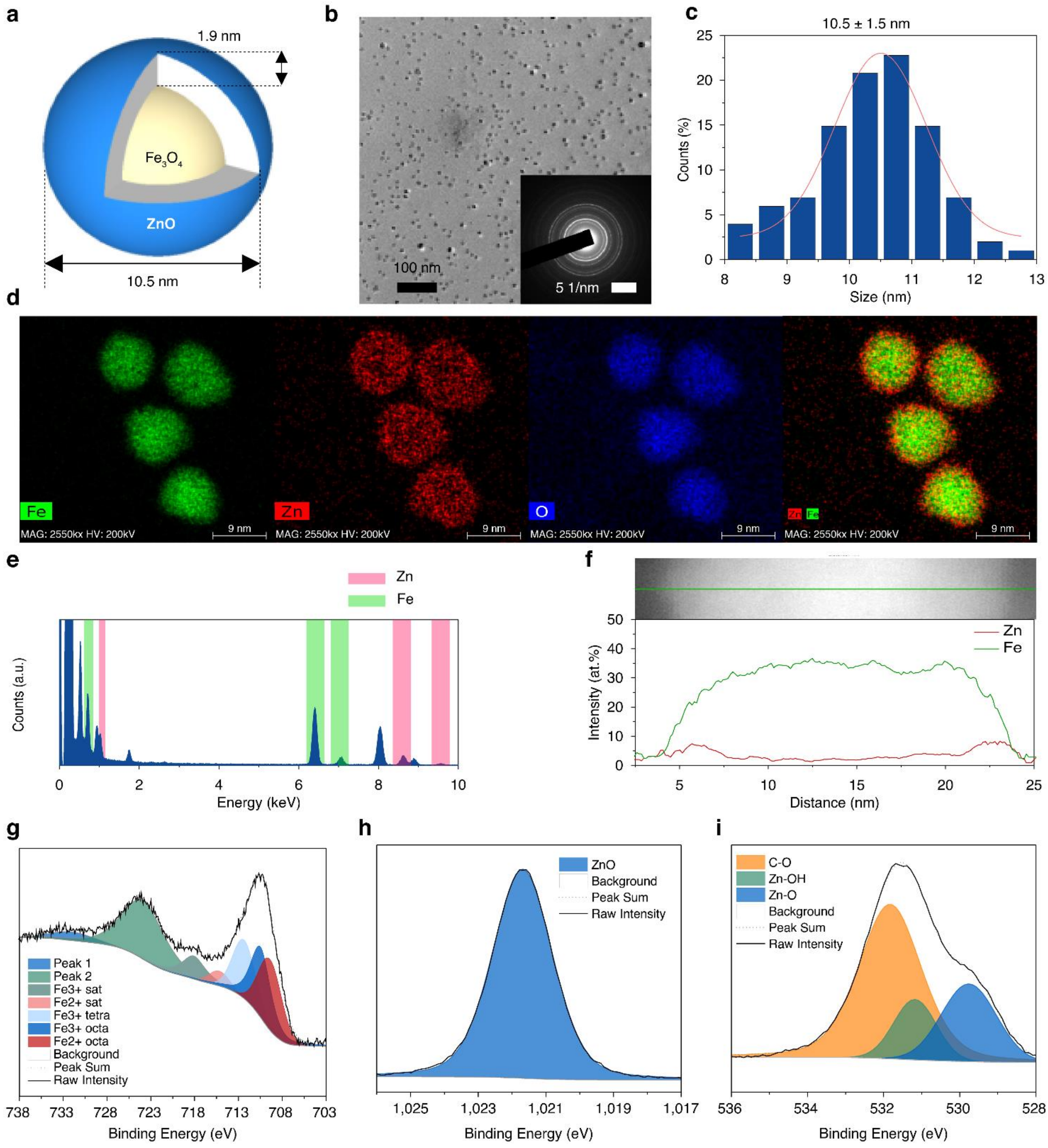

Figure S8. Characteristics of Fe_3O_4-ZnO core-shell nanoparticle. a) Diagram of the Fe_3O_4-ZnO core-shell nanoparticle. b) TEM image and SAED pattern of Fe_3O_4-ZnO core-shell nanoparticle. c) Size distribution of Fe_3O_4-ZnO core-shell nanoparticle. The mean value of diameter was obtained by fitting the counts using Gaussian function. d) Elemental mapping images of Fe_3O_4-ZnO core-shell

nanoparticle; Fe (green), Zn (red), Oxygen (blue). e) Point-probe analysis of EDS that collected the signal from a single nanoparticle. f) Energy Dispersive Spectrometry (EDS) line scan of the elemental composition; Fe (green), Zn (red). The boundary between ZnO and Fe_3O_4 is distinguished and ZnO covers the entire surface of the Fe3O4 core. In particular, the thickness of ZnO is approximately 10% of the core size, forming a 1.9 nm-layer. g–i) X-ray photoelectron spectra and deconvoluted peaks of Fe_3O_4-ZnO core-shell nanoparticle at different energy levels: g) Fe 2p; h) Zn 2p; i) O 1s. The Fe^{2+}/Fe^{3+} ratio derived from the Fe $2p_{3/2}$ transition in Fe 2*p* XPS was estimated at 0.5, which was in good agreement with stoichiometry of Fe_3O_4. The peak at 1021.7 eV in Zn 2*p* XPS originated from the formation of the ZnO phase rather than the $Zn(OH)_2$ phase.

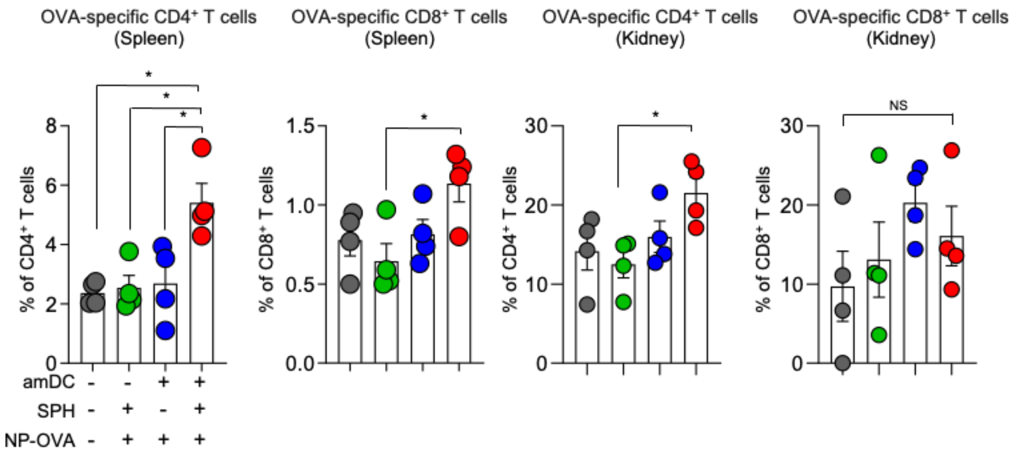

Figure S9. SVF SPH and antigen-loaded amDCs enhance antigen-specific T cell response in both systemic and local immunity. Frequency of OVA-specific $CD4^+$ and $CD8^+$ T cells in the indicated groups, in the spleen and kidney, representing systemic and local immunity (n = 4, mean ± s.e.m.). *: $p < 0.05$, NS, non-significant using one-way ANOVA with Tukey's post-test; SVF SPHs, stromal vascular fraction cell spheroids; amDCs, NP-OVA-loaded mature dendritic cells; OVA, ovalbumin.

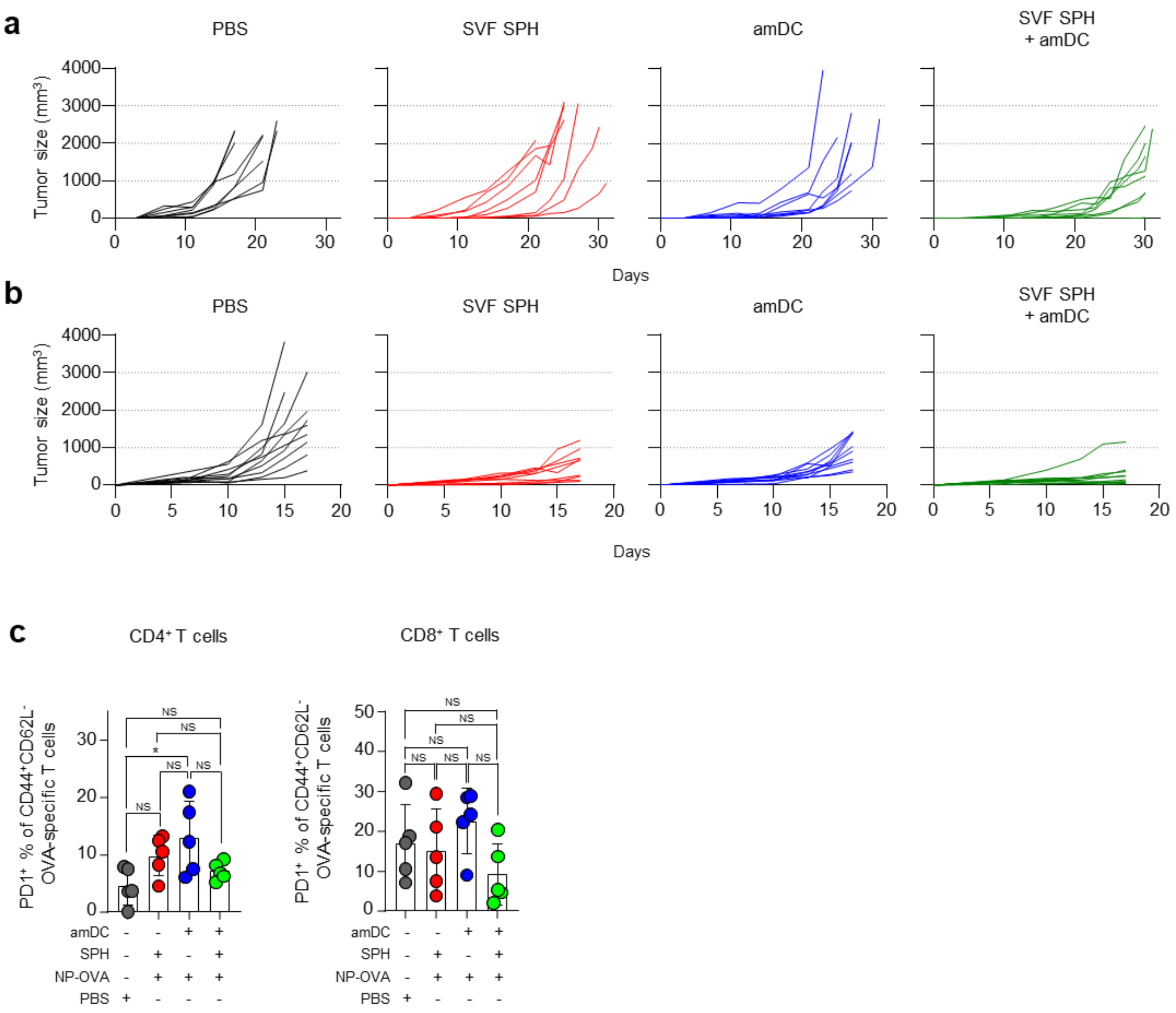

Figure S10. Evaluation of the anti-tumor effect generated by SVF SPH and antigen-pulsed amDCs. a) Tumor progression curves of individual mice shown in Figure 6a-c (*n* = 8/group*)*. b) Tumor progression curves of individual mice shown in Figure 6d-h (*n* = 9 or 10). c) Frequency of PD-1$^+$ cells in CD44$^+$ CD62$^-$ effector T cells in the indicated groups (*n* = 5, mean ± s.e.m.). *: $p < 0.05$, **: $p < 0.01$, ***: $p < 0.001$, NS, non-significant by one-way ANOVA with Tukey's post-test; SVF SPH, stromal vascular fraction cell spheroids; amDC, NP-OVA-loaded dendritic cells

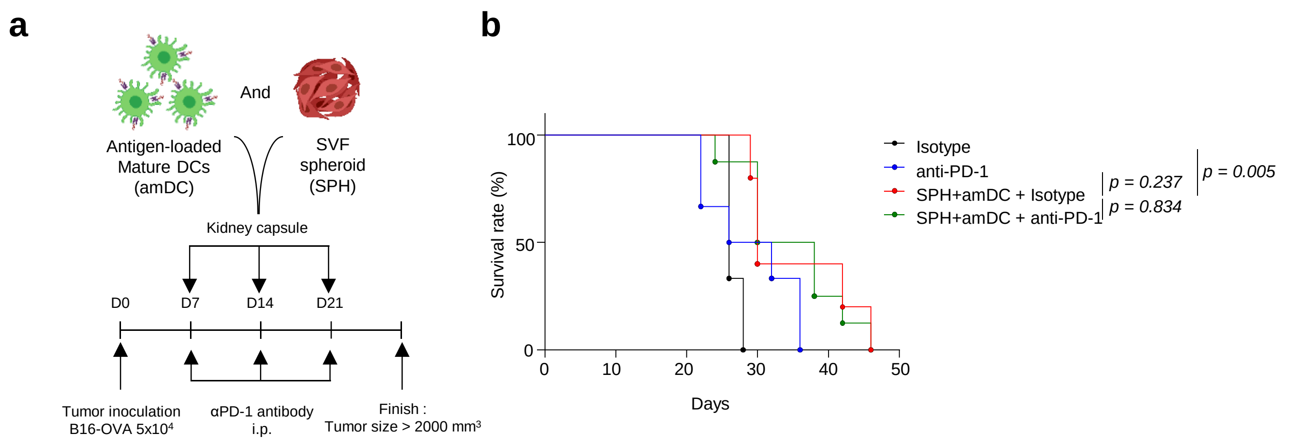

Figure S11. Evaluation of anti-tumor effect generated by combination therapy of SVF SPH and antigen-pulsed amDCs with anti-PD-1 therapy. a) Experimental scheme for combination therapy of immunization with SVF SPHs and amDCs in mice bearing a tumor (OVA-expressing B16 melanoma) via sub-renal capsule transplantation and intraperitoneal injection (i.p.) of anti-PD-1 therapy. b) Survival curves (*n* = 3-8/group). Mantel-Cox text; SPH, stromal vascular fraction cell spheroids; amDC, NP-OVA-loaded dendritic cells

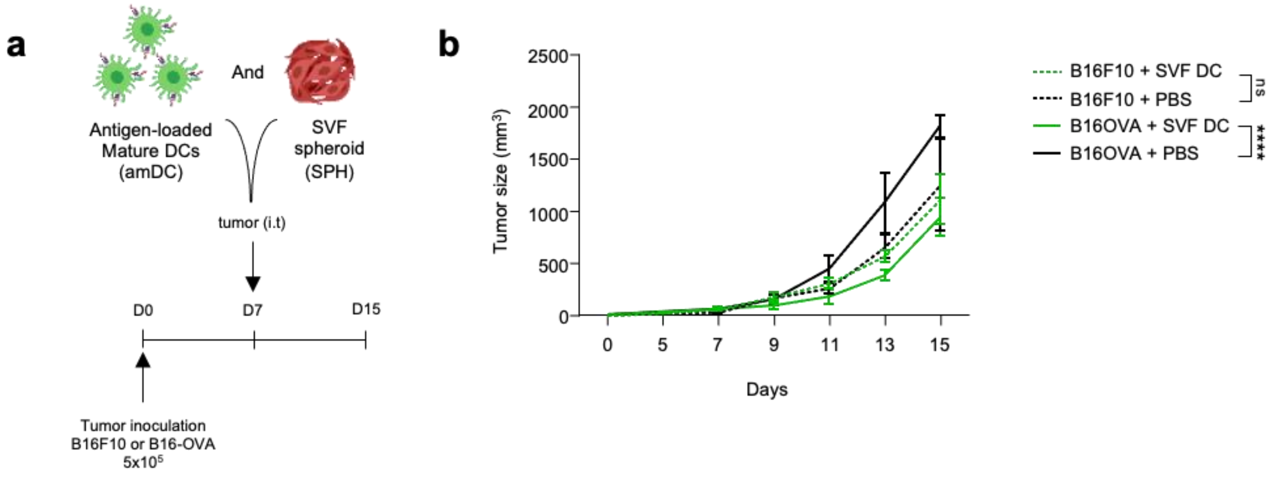

Figure S12. Evaluation of antigen specific anti-tumor responses of SVF SPHs and amDCs. a) Experimental scheme for intratumoral injection (i.t.) of SVF SPHs and amDCs in mice bearing a tumor (OVA-expressing B16 melanoma and B16F10 control). b) Tumor progression (*n* = 5-6/group, mean ± s.e.m.). ****: $p < 0.0001$; NS, non-significant using two-way ANOVA with Tukey's post-test (b).

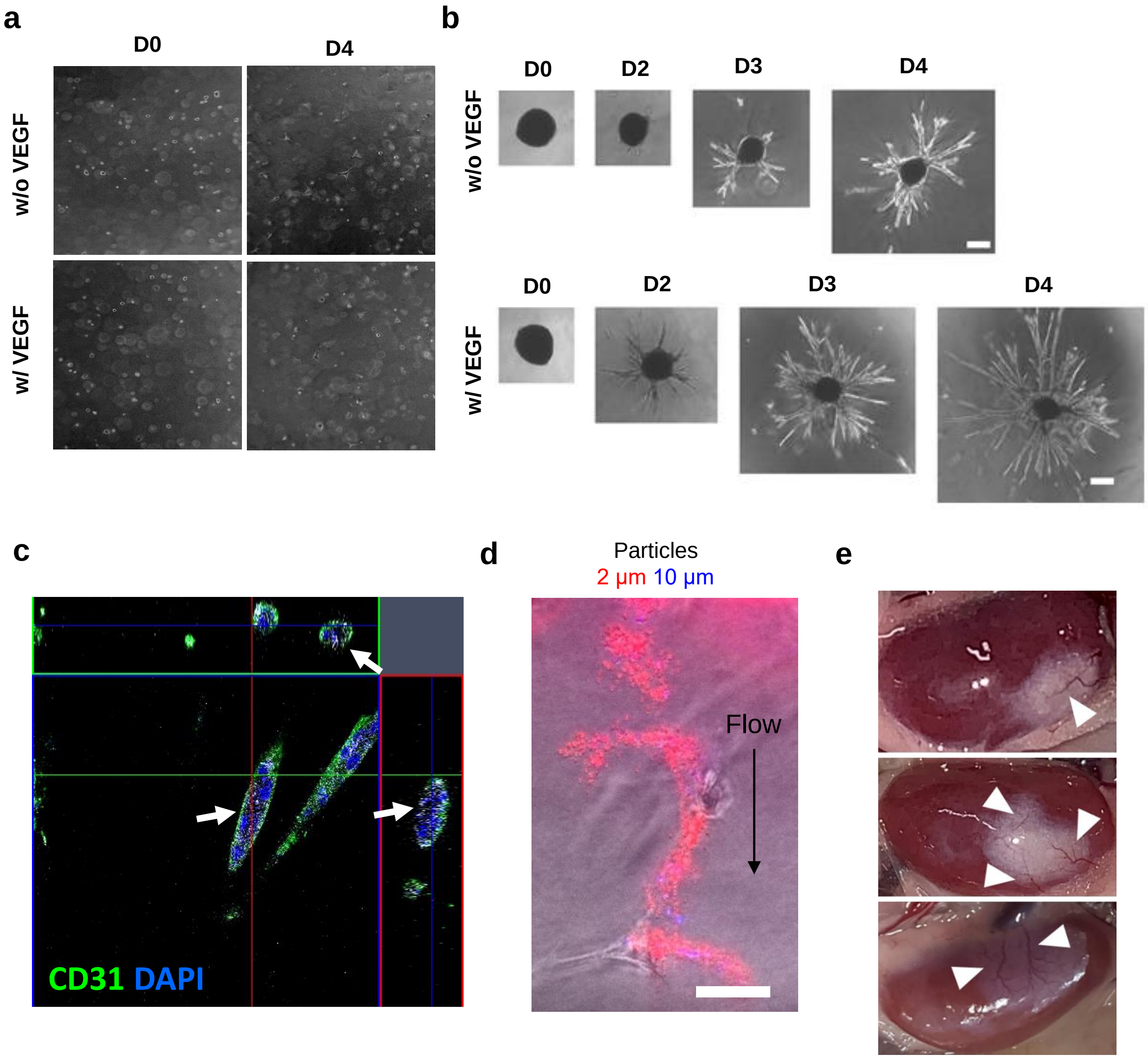

Figure S13. SVF SPH generate vessel-like structures. a-d) Single cells (2D) or spheroids (3D) of SVF cells were incubated in 3 mg/mL collagen type I with or without 100 ng/mL of VEGF-A and -C contained medium for indicated dates. a) Regardless of VEGF treatment, SVF cells cultured in 2D did not develop any structure. b) SVF cells cultured in 3D (SVF SPH) generated sprouts on day 2 with VEGF treatment or on day 3 without VEGF treatment. Scale bar: 200 µm. c) Sprouts had a vacant hole at the core indicated by white arrows in the section image. d) 2 µm (red) and 10 µm (blue) of particles were passed through this hole within the sprouts from SVF SPH cultured in

collagen for 3 days. The fluid flow ran from top to bottom (direction indicated with black arrow). Scale bar: 100 μm. e) Photographic images of blood vessels on the surface of the grafts (white arrowheads).

Movie S1. Cell migration assay to assess the chemotactic effect of SVF SPHs for 24 h. Negative control. Left: no cells, right: splenocytes.

Movie S2. Cell migration assay to assess the chemotactic effect of SVF SPHs for 24 h. Experimental test. Left: SVF SPH, right: splenocytes.
